# Supplementary material for: Seroprevalence of viral and vector-borne bacterial pathogens in domestic dogs (Canis familiaris) in northern Botswana
Source: PLoS One. 2020 Jan 8;15(1):e0220593. doi: 10.1371/journal.pone.0220593 (PMC6949005; doi:10.1371/journal.pone.0220593)
Supplement: Attachment — Submitted filename: Review Result for PONE.pdf [file pone.0220593.s002.pdf]

Review Result for PONE-D-19-20072

Title: Adequate

Suggestion for Short Title: Prevalence of antibodies to infectious agents in Botswanan dogs

Instead of Infectious disease prevalence in dogs in Botswana since seroprevalence does not indicate active infection

Abstract

Ehrlichia spp, *Anaplasma* spp.- spp should not be italicized. See examples in lines 30, 38, 40, 45 and in fact throughout the text and tables: Please correct them appropriately.

Line 36- delete "total of" from the sentence

Conclusion: Authors hinged their conclusion on tick control and -------. However, there was no mention of tick infestation status/ type of ticks on the screened population nor tick control practices by the dog owners. This aspect should be stressed in the methodology to justify the recommendation of increasing tick control to promote health of dogs and wildlife. Otherwise downplay the aspect of tick control since tick infestation status of the dogs was not assessed in the study.

Introduction

Line 59 and several others: shouldn't it be [3-5] instead of [3,4,5]? Refer to journal style

Line 59-60 suggested to modify as follows: Capturing a sufficient number of these wild canids and felids to perform seroprevalence surveys is not always feasible due to the risk associated with anesthesia necessary to collect blood samples from them instead of Capturing a sufficient number of African wild dogs, black-footed cats, cheetah, or lion to perform seroprevalence surveys is not always feasible due to the risk associated with anesthesia necessary to collect blood samples from these animals.

Line 63: McRee, et al. [6] performed a seroprevalence study of -----

Line 66: see previous comment on spp.,

Line 67: specify the tick vector for each of the pathogens mentioned.

Lines 84-86 Therefore, the objective of this study was to assess the seroprevalence of common infectious diseases (Ehrlichia spp., Borrelia burgdorferi, Anaplasma spp., Dirofilaria immitis, CDV, CAV and CPV)……What you have in the bracket are pathogens and not diseases. Rephrase to the name of the disease and not the agent eg Ehrlichiosis, Borellioisis, ------- or change diseases in the sentence to agents.

Why did the authors not include rabies in the screening tests considering the role of domestic dogs and wildlife in the epidemiology of the disease?

Materials and methods

There is no information on tick infestation at time of sampling and control measures being used by the owners. Authors did not mentioned/describe how demographic data such age, sex, breed, vaccination status of dogs, history of travel, medical/vaccination history, the use of dogs either pet, security or hunting etc of the dogs were obtained. This is vital to the risk assessment aspect of the study

Lines 100-101- Did the authors conducted the test on whole blood, plasma or serum? If not blood, they should explain how what was used was obtained.

Serological Analyses

Lines 104-110. Which antibody type; IgG or IgM was being determined by the serological kits used by the authors. What are the specificities and sensitives of these kits? How did the authors differentiate between antibodies due to vaccination and natural infections especially for CD, CA and CP infections in the vaccinated population since they do not have history of importation or travel outside Botswana of the study population?

The interpretation of results based on visual color change could be subjective, how did the authors ensured that the results were not biased. What did they consider to be their positive in case of the test where results could be either positive, highly positive or inconclusive reactions and what is the implication of these variation in the reactions? Could it be titer level or active and passive infection etc? please explain

Statistical analyses- mention the statistical package/software used for the analysis

Results

Lines 125-126: How was the age of the dogs determined. What criteria or age range was used to classify dogs as adult or juveniles?

Line 134: is tit 232 or 233?

Table 2: I am not sure the authors explained why there are two populations; 232 and 233 being tested for different pathogens

Table 3: The table can be presented in a better format without having to repeat the variables in columns 1 and 2 for the two pathogens. Why are some variables in column 1 in italics? (see throughout the tables)

Tables 4 and 5 should be merged similar to comments made for table 3 to avoid duplicating the variables in columns 1 and 2.

Discussion

Lines 196-200. Rephrase the sentence starting from " A survey of domestic dogs and African Wild dogs in……. to E. canis [21], there are too many repetitions.

Lines 220-221. Lack of data on tick infestation in this study limits the strength of your discussion on these pathogens.

Lines 229-231: But we do not know the infestation rate in the domestic dog population to draw any meaningful inference.

Line 240-41: What could influence the significant association of CDV with June than July? The study duration of 2 months to me is too short to make any meaningful deduction other than chance findings.

References

Reference 10. Author names are written in uppercase. Correct

Genus and species names of pathogens and animals were not italicized in several references. Please correct them according to the accepted standard.
