# Supplementary material for: Seroprevalence of viral and vector-borne bacterial pathogens in domestic dogs (Canis familiaris) in northern Botswana
Source: PLoS One. 2020 Jan 8;15(1):e0220593. doi: 10.1371/journal.pone.0220593 (PMC6949005; doi:10.1371/journal.pone.0220593)
Supplement: Attachment — Submitted filename: Cor PONE-D-19-20072 R1.pdf [file pone.0220593.s003.pdf]

PLOS ONE

Seroprevalence of viral and vector-borne bacterial pathogens in domestic dogs (Canis familiaris) in northern Botswana

--Manuscript Draft--

**Manuscript Number:**	PONE-D-19-20072R1
**Article Type:**	Research Article
**Full Title:**	Seroprevalence of viral and vector-borne bacterial pathogens in domestic dogs (Canis familiaris) in northern Botswana
**Short Title:**	Prevalence of antibodies to infectious agents in Botswanan dogs
**Corresponding Author:**	Melissa Kennedy<br>University of Tennessee<br>Alcoa, TN UNITED STATES
**Keywords:**	Epidemiology; domestic dogs; Wildlife; Botswana; infectious agents
**Abstract:**	Abstract<br>Background: Domestic dogs (Canis familiaris) have the potential to act as disease reservoirs for wildlife and are important sentinels for common circulating pathogens. Therefore, the infectious agent seroprevalence among domestic dogs in northern Botswana may be indicative of pathogen exposure of various wildlife species. The objective of this study was to assess the seroprevalence of Ehrlichia spp., Borrelia burgdorferi , Anaplasma spp . , Dirofilaria immitis , canine adenovirus, canine parvovirus-2, and canine distemper virus in domestic dogs as proxies of disease prevalence in the local wildlife in the Okavango Delta region of Botswana. Statistical analysis assessed crude and factor-specific seroprevalence proportions and assessed the factors age, sex, and geographical location as predictors of seropositivity. Logistic regression was used to identify adjusted predictors of seropositivity for each of the pathogens of interest.<br>Results: Samples from 233 dogs in 7 locations in Maun, Botswana, and surrounding villages were collected and serologically analyzed. No dogs were seropositive for B. burgdorferi, while low seroprevalence proportions were observed for Anaplasma spp . (2.2%) and D. immitis (0.9%). Higher seroprevalence proportions were observed for the tick-borne pathogen, Ehrlichia spp . (21.0%), and 19.7% were seropositive for canine adenovirus (hepatitis) . The highest seroprevalence proportions were for canine parvovirus-2 (70.0%) and canine distemper virus (44.8%). The predictors of seropositivity revealed that adults were more likely to be seropositive for canine adenovirus, canine distemper virus, and canine parvovirus-2 than juveniles, and location was a risk factor for canine adenovirus, canine distemper virus, canine parvovirus-2, and Ehrlichia spp.<br>Conclusions: Results indicate a need for tick prevalence data and that increasing vaccination campaigns for domestic dogs may improve the health of domestic animals, and potentially wildlife and humans in the Okavango Delta since viral and vector-borne bacterial pathogens can be transmitted between them.
**Order of Authors:**	Riley E Thompson
	Hayley Adams
	Agricola Odoi
	Melissa Kennedy
**Response to Reviewers:**	Reviewers' comments:<br>Laboratory methods – given that the only techniques used involved ELISA kits per manufacturers' recommendation, none were deposited.<br>The style requirements were reviewed.<br>Owners' verbal consent for participation, the locale of participants, and the volume of blood collected were addressed in the text.<br>Reviewer #1:<br>The short title was modified per the reviewer's recommendations.<br>The italics for "spp." designations were removed.<br>"total of" was removed from the sentence in line 37.

Powered by Editorial Manager® and ProduXion Manager® from Aries Systems Corporation

	The issue of tick infestation and control were addressed in the text<br>Lines 60-61 and line 64 were modified per the reviewer's recommendations.<br>The tick vector for each organism was included.<br>Rephrasing of lines 87-89 was made.<br>As stated in the manuscript, rabies was not assessed because of unknown vaccination status for rabies, and the lack of a point-of-care assay for this assessment.<br>The various aspects of demographic data were addressed in the text.<br>The use of whole blood was addressed<br>The various properties of the kits were addressed.<br>Color change evaluation was addressed.<br>Tables were condensed.<br>Lines 202-206 were modified per the reviewer's recommendation.<br>The reference corrections were made.<br><br><br>Reviewer #2:<br>The hypothesis, objectives and methods were clarified.<br>Demographic data was addressed.<br>The discussion section was adjusted.<br>Tables were reduced:<br><br>Reviewer Comment<br>Line 134: is it 232 or 233?<br><br>Response<br>The total sample size was 233. However, there was one missing data point for test results of the following pathogens: Anaplasma spp., B.burgdorferi and D.immitis. Due to that missing data, these pathogens had 232 instead of 233. The rest of the pathogens did not have missing data and therefore had a total of 233. These explanations have been added as footnotes on tables 2, 3a and 3b<br><br>Reviewer Comment<br>Table 2: I am not sure the authors explained why there are two populations; 232 233 being tested for different pathogens.<br><br>Response<br>There is only one population of dogs. However, as explained above there was there was one missing data point for test results for Anaplasma spp., B.burgdorferi and D.immitis. Therefore these pathogens had data for only 232 dogs instead of 233 that the rest of the pathogen had.<br><br>Reviewer Comment<br>Table 3: The table can be presented in a better format without having to repeat the variables in columns 1 and 2 for the two pathogens. Why are some variables in column 1 in italics? (see throughout the tables)<br><br>Response<br>We thank the reviewer for their suggestion on improving the presentation of our results. As per the suggestion, tables 3, 4 and 5 have now been combined into Table 3. However, since the new Table 3 cannot fit on one page, it has been split into Table 3a and Table 3b. This change has allowed us to stop repeating information from column 1. However, we have retained column 2 because the data changes between pathogens depending on whether there was missing data or not.<br><br>Reviewer Comment<br>Tables 4 and 5 should be merged similar to comments made for table 3 to avoid duplicating the variables in columns 1 and 2.<br><br>Response:<br>As stated above tables 3, 4 and 5 have not been combined into Table 3. Since the Table 3 cannot fit on one page, it has been split into Table 3a and Table 3b. This change has allowed us to stop repeating information from column 1. However, we have retained column 2 because the data changes between pathogens depending on whether there was missing data or not.

Powered by Editorial Manager® and ProduXion Manager® from Aries Systems Corporation

	Reviewer Comment<br>Line 240-41: What could influence the significant association of CDV with June than July? The study duration of 2 months to me is too short to make any meaningful deduction other than chance findings.<br><br>Response:<br>We thank the reviewer for noting this association and seeking an explanation. Unfortunately, this being a sero-survey, the scope of the study was limited and so we were not able to investigate this association further. However, due to the very strong association (OR = 7.8; p=0.002), it is unlikely to be due to chance. Suffice it to say that future studies will need to investigate this association further. We have added this comment in the manuscript.<br><br>Reference corrections were made.
**Additional Information:**	
**Question**	**Response**
**Financial Disclosure**<br><br>Enter a financial disclosure statement that describes the sources of funding for the work included in this submission. Review the submission guidelines for detailed requirements. View published research articles from *PLOS ONE* for specific examples.<br><br>This statement is required for submission and **will appear in the published article** if the submission is accepted. Please make sure it is accurate.<br><br>**Unfunded studies**<br>Enter: *The author(s) received no specific funding for this work.*<br><br>**Funded studies**<br>Enter a statement with the following details:<br>• Initials of the authors who received each award<br>• Grant numbers awarded to each author<br>• The full name of each funder<br>• URL of each funder website<br>• Did the sponsors or funders play any role in the study design, data collection and analysis, decision to publish, or preparation of the manuscript?<br>• **NO** - Include this sentence at the end of your statement: *The funders had no role in study design, data collection and analysis, decision to publish, or preparation of the manuscript.*<br>• **YES** - Specify the role(s) played.	MAK<br>Grant #D16CA-605<br>Morris Animal Foundation Veterinary Student Scholars<br>https://www.morrisanimalfoundation.org/sites/default/files/filesync/VSS-Guidelines.pdf<br>The funders had no role in study design, data collection and analysis, decision to publish, or preparation of the manuscript.

Powered by Editorial Manager® and ProduXion Manager® from Aries Systems Corporation

* typeset	
**Competing Interests**<br><br>Use the instructions below to enter a competing interest statement for this submission. On behalf of all authors, disclose any competing interests that could be perceived to bias this work—acknowledging all financial support and any other relevant financial or non-financial competing interests.<br><br>This statement **will appear in the published article** if the submission is accepted. Please make sure it is accurate. View published research articles from *PLOS ONE* for specific examples.<br><br>**NO authors have competing interests**<br><br>Enter: *The authors have declared that no competing interests exist.*<br><br>**Authors with competing interests**<br><br>Enter competing interest details beginning with this statement:<br><br>*I have read the journal's policy and the authors of this manuscript have the following competing interests: [insert competing interests here]*<br><br>* typeset	The authors have declared that no competing interests exist.
**Ethics Statement**<br><br>Enter an ethics statement for this submission. This statement is required if the study involved:<br><br>• Human participants<br>• Human specimens or tissue<br>• Vertebrate animals or cephalopods<br>• Vertebrate embryos or tissues<br>• Field research<br><br>Write "N/A" if the submission does not require an ethics statement.	The University of Tennessee Knoxville Institutional Animal Care and Use Committee approved this research proposal March 27, 2015; #2333-0315.

General guidance is provided below. Consult the submission guidelines for detailed instructions. **Make sure that all information entered here is included in the Methods section of the manuscript.**<br><br>**Format for specific study types**<br><br>**Human Subject Research (involving human participants and/or tissue)**<br>• Give the name of the institutional review board or ethics committee that approved the study<br>• Include the approval number and/or a statement indicating approval of this research<br>• Indicate the form of consent obtained (written/oral) or the reason that consent was not obtained (e.g. the data were analyzed anonymously)<br><br>**Animal Research (involving vertebrate animals, embryos or tissues)**<br>• Provide the name of the Institutional Animal Care and Use Committee (IACUC) or other relevant ethics board that reviewed the study protocol, and indicate whether they approved this research or granted a formal waiver of ethical approval<br>• Include an approval number if one was obtained<br>• If the study involved *non-human primates*, add *additional details* about animal welfare and steps taken to ameliorate suffering<br>• If anesthesia, euthanasia, or any kind of animal sacrifice is part of the study, include briefly which substances and/or methods were applied<br><br>**Field Research**<br><br>Include the following details if this study involves the collection of plant, animal, or other materials from a natural setting:<br>• Field permit number<br>• Name of the institution or relevant body that granted permission	
**Data Availability**<br><br>Authors are required to make all data underlying the findings described fully	Yes - all data are fully available without restriction

Powered by Editorial Manager® and ProduXion Manager® from Aries Systems Corporation

available, without restriction, and from the time of publication. PLOS allows rare exceptions to address legal and ethical concerns. See the PLOS Data Policy and FAQ for detailed information.<br><br>A Data Availability Statement describing where the data can be found is required at submission. Your answers to this question constitute the Data Availability Statement and **will be published in the article**, if accepted.<br><br>**Important:** Stating 'data available on request from the author' is not sufficient. If your data are only available upon request, select 'No' for the first question and explain your exceptional situation in the text box.<br><br>Do the authors confirm that all data underlying the findings described in their manuscript are fully available without restriction?	
**Describe where the data may be found in full sentences. If you are copying our sample text, replace any instances of XXX with the appropriate details.**<br><br>• If the data are **held or will be held in a public repository**, include URLs, accession numbers or DOIs. If this information will only be available after acceptance, indicate this by ticking the box below. For example: *All XXX files are available from the XXX database (accession number(s) XXX, XXX.*).<br>• If the data are all contained **within the manuscript and/or Supporting Information files**, enter the following: *All relevant data are within the manuscript and its Supporting Information files.*<br>• If neither of these applies but you are able to provide **details of access elsewhere**, with or without limitations, please do so. For example:<br><br>*Data cannot be shared publicly because of [XXX]. Data are available from the XXX Institutional Data Access / Ethics*	All relevant data are within the manuscript and its Supporting Information files.

Committee (contact via XXX) for researchers who meet the criteria for access to confidential data.<br><br>*The data underlying the results presented in the study are available from (include the name of the third party and contact information or URL).*<br>• This text is appropriate if the data are owned by a third party and authors do not have permission to share the data.<br><br>* typeset	
Additional data availability information:	

Powered by Editorial Manager® and ProduXion Manager® from Aries Systems Corporation

Manuscript
Click here to access/download;Manuscript;Botswana manuscript PLOS submission.docx

Seroprevalence of viral and vector-borne bacterial pathogens in domestic dogs (*Canis familiaris)* in northern Botswana

Short title: Infectious disease prevalence in dogs in Botswana

Riley E. Thompson[1], Hayley Adams[2], Agricola Odoi[3], Melissa A. Kennedy[3]

[1]Department of Large Animal Clinical Sciences, College of Veterinary Medicine, University of Tennessee, Knoxville, TN, USA, [2]Silent Heroes Foundation, USA, [3]Department of Biomedical and Diagnostic Sciences, College of Veterinary Medicine, University of Tennessee, Knoxville, TN, USA

Correspondence to:

Melissa Kennedy

Email:

mkenned2@utk.edu

Author contributions

R.E.T. (University of Tennessee) collected samples, performed ELISA testing, and was involved in manuscript writing and edits, H.A. (Silent Heroes Foundation) designed experiments and arranged local logistics, A.O. (University of Tennessee) performed statistical analysis and was involved in manuscript writing and edits, and M.A.K. (University of Tennessee) was the project leader and was responsible for experimental design and data analysis.

Abstract

Background: Domestic dogs (*Canis familiaris*) have the potential to act as disease reservoirs for wildlife and are important sentinels for common circulating pathogens. Therefore, the infectious disease seroprevalence among domestic dogs in northern Botswana may be indicative of pathogen exposure of various wildlife species. The objective of this study was to assess the seroprevalence of *Ehrlichia spp.*, *Borrelia burgdorferi*, *Anaplasma spp.*, *Dirofilaria immitis*, canine adenovirus, canine parvovirus, and canine distemper virus in domestic dogs as proxies of disease prevalence in the local wildlife in the Okavango Delta region of Botswana. Statistical analysis assessed crude and factor-specific seroprevalence proportions and assessed the factors age, sex, and geographical location as predictors of seropositivity. Logistic regression was used to identify adjusted predictors of seropositivity for each of the pathogens of interest.

Results: Samples from 233 dogs in a total of 7 locations in Maun, Botswana, and surrounding villages were collected and serologically analyzed. No dogs were seropositive for *B. burgdorferi,* while low seroprevalence proportions were observed for *Anaplasma spp.* (2.2%) and *D. immitis* (0.9%). Higher seroprevalence proportions were observed for the tick-borne pathogen, *Ehrlichia spp.* (21.0%), and 19.7% were seropositive for canine adenovirus (hepatitis). The highest seroprevalence proportions were for canine parvovirus (70.0%) and canine distemper virus (44.8%). The predictors of seropositivity revealed that adults were more likely to be seropositive for canine adenovirus, canine distemper virus, and canine parvovirus than juveniles, and location was a risk factor for canine adenovirus, canine distemper virus, canine parvovirus, and *Ehrlichia spp*.

Conclusions: Results indicate that increasing tick control and vaccination campaigns for domestic dogs may improve the health of domestic animals, and potentially wildlife and humans in the Okavango Delta since viral and vector-borne bacterial pathogens can be transmitted between them.

Introduction

Vaccination of domestic dogs has been reported as a method of wildlife conservation [1] with the implication that prevalence of transmissible diseases in the domestic canine population has the potential to affect disease burden in wildlife, including both wild felids and wild canids. African wild dogs (*Lycaon pictus*), a wild canid species in sub-Saharan Africa, are endangered according to the International Union for the Conservation of Nature Redlist [2], and the black-footed cat (*Felis nigripes*), cheetah (*Acinonyx jubatus*), and lion (*Panthera leo*) are all vulnerable species in Botswana [2] that can be negatively impacted by domestic dog viral and vector-borne bacterial pathogens [3, 4, 5]. Capturing a sufficient number of African wild dogs, black-footed cats, cheetah, or lion to perform seroprevalence surveys is not always feasible due to the risk associated with anesthesia necessary to collect blood samples from these animals. This is particularly concerning due to the low numbers of individuals as indicated by their conservation status. McRee, et al. [6] performed a prevalence evaluation of viral pathogens in domestic dogs in northwest Zimbabwe as a representation of wildlife viral disease prevalence in the region, particularly African wild dogs.

Ehrlichia spp., *Anaplasma spp*., and *Borrelia burgdorferi* (Lyme disease) are bacterial pathogens that are transmitted by tick bites [7, 8, 9]. *Dirofilaria immitis*, or heartworm disease, is a blood-borne parasite transmitted by mosquito bites [10], and canine distemper (CDV),

parvovirus (CPV), and adenovirus (CAV) are viral diseases transmitted between individuals [11, 12, 13]. All of these common pathogens in dogs can cause serious illness and/or death. While the viral diseases, Lyme disease, and heartworm disease can be prevented either by vaccination or monthly heartworm preventative medication, many communities in southern Africa do not have the resources to pay for these medications for their animals. Thus, these preventable diseases may be widespread.

Botswana is a land-locked country in southern Africa and is home to the Okavango Delta, a diverse wetland habitat. Not only is the Okavango Delta home to countless species, it is the center for tourism in the country, which has become the second most important industry in Botswana after diamond mining [14]. The Okavango Delta of Botswana is rich with wildlife which have the chance to interact with domestic animal populations. This potentially results in cross-transmission of infections between domestic and wild animals implying that infectious disease exposure in domestic animals might mirror those of wildlife. As disease prevalence of common infectious diseases in wild carnivores is unknown in the Okavango Delta, this presents the opportunity to use domestic dogs as sentinels for infectious disease exposure in wildlife. Therefore, the objective of this study was to assess the seroprevalence of common infectious diseases (*Ehrlichia spp.*, *Borrelia burgdorferi*, *Anaplasma spp.*, *Dirofilaria immitis,* CDV, CAV, and CPV) in domestic dogs in Maun, Botswana, an area adjacent to the Okavango Delta, as a proxy for seroprevalence that would be expected in the wild canid and felid populations.

Materials and Methods

Animals

All blood collections were done under the direct supervision of the veterinary members of the Maun Animal Welfare Society (MAWS). The majority (n=128/233) of blood samples from domestic dogs were collected using a convenience sampling strategy at the MAWS in Maun, Botswana, from dogs who were presented for castration and vaccination. The remaining blood samples (n=105/233) were collected in surrounding villages. The uncastrated domestic dogs in this area were highly unlikely to have been vaccinated and be seropositive to the viral diseases due to vaccination cross-reaction because MAWS is the main veterinary clinic in the area for the low income population, and they will not vaccinate animals unless they are also castrated at the time of vaccination. Therefore, animals that were reproductively intact are likely to be unvaccinated. Blood samples were collected from a peripheral vein, transported on ice, and stored at -20°C until testing.

Sample Analyses

Seroprevalence for CAV, CPV, and CDV were assessed using Biogal Titer Check (Biogal Galed Laboratories, Kibbutz Galed, Israel) per manufacturer instructions. The Biogal Titer Check results were reported with a change of color and 'negative', 'positive', 'highly positive', or 'inconclusive' as the range of possible results. The vector-borne diseases, *Ehrlichia spp.*, *Borrelia burgdorferi*, *Anaplasma spp.*, and *Dirofilaria immitis*, were assessed with IDEXX 4DX SNAP ELISA (IDEXX, Westbrook, Maine, USA) per manufacturer instructions. The IDEXX SNAP ELISA results were reported as either 'positive' or 'negative' by a color change.

Statistical Analysis

Crude and factor-specific seroprevalence proportions of *Ehrlichia spp.*, *Borrelia burgdorferi*, *Anaplasma spp.*, *Dirofilaria immitis*, CDV, CAV and CPV as well as their 95% exact confidence intervals were computed. The factors considered were age, sex, and location. Associations between seroprevalence and each of the above factors were assessed using the Chi-square or Fishers Exact tests as appropriate. Significance was set at $\alpha = 0.05$ for all statistical tests. Logistic regression was used to identify adjusted predictors of seropositivity for each of the pathogens of interest.

Results

Animal demographics

A total of 233 dogs were tested (Table 1). Females dogs made up the majority (54.5%) of the sampled dogs. Dogs were sampled during the months of June and July 2015 with the majority (55.4%) of the dogs being sampled in July. The age group distribution was 69.7% adults and 30.0% juveniles. Samples were collected at seven locations in Maun, Botswana, and surrounding villages: MAWS (n=128), Khumaga (n=13), Boro (n=18), Mathapane (n=16), Shorobe (n=18), Sexaxa (n=8), and Etsha (n=32).

Table 1: Characteristics of dogs included in a seroprevalence assessment of prior exposure to common pathogens in Botswana, 2015

Variable	Number	Percent	95% Exact Binomial Confidence Interval
Sex			
Female	127	54.5	47.9, 61.0
Male	106	45.5	3.90, 52.1
Age Category			
Adult	163	69.7	63.6, 75.8
Juvenile	70	30.0	24.2, 36.3

Location			
Boro	18	7.7	4.6, 11.9
Etsha	32	13.7	9.6, 18.8
Khumaga	13	5.6	3.0, 9.4
MAWS	128	54.9	48.3, 61.4
Mathapane	16	6.9	4.0, 10.9
Sexaxa	8	3.4	1.5, 6.7
Shorobe	18	7.7	4.6, 11.9
Month			
June 2015	104	44.6	38.1, 51.0
July 2015	129	55.4	48.7, 61.8

Crude seroprevalence

Of 232 individuals, 0% were seropositive for *Borrelia burgdorferi*, 2.2% were seropositive for *Anaplasma spp.*, and 0.9% were seropositive for *Dirofilaria immitis* (Table 2). Out of the 233 animals, 21.0% were seropositive for *Ehrlichia spp.*, 19.7% were seropositive for CAV, 70% were seropositive for CPV, and 46.8% were seropositive for CDV.

Table 2: Crude seroprevalence of selected pathogens among domestic dogs in Botswana, 2015

Pathogen	n	Number of seropositive samples	Percentage of seropositive samples	95% Confidence Interval
Anaplasma spp.	232	5	2.2	0.7, 5.0
B.burgdorferi	232	0	0	0, 1.6
D.immitis	232	2	0.9	0.1, 3.1
CDV	233	109	46.8	40.2, 53.4
Ehrlichia spp.	233	49	21.0	16.0, 26.8
CAV	233	46	19.7	14.8, 24.4
CPV	233	163	70.0	63.6, 75.8

Predictors of seropositivity

Based on the results of the logistic model, there were no statistically significant predictors (sex, age, month, or location) (P>0.05) for seropositivity of either *Anaplasma spp.* or *D. immitus* (Table 3).

Although age of the dog and location had significant unadjusted associations with the odds of CAV seropositivity, only age (OR=4.4; p<0.0003) was significant in the final; implying that the odds of CAV seropositivity was 4.4 times higher in adults dogs than in juveniles (Table 4). Similarly, only age had a significant association in the final model for CPV with adults having 4.4 times higher odds of CPV seropositivity (OR 4.4; p<0.0001) than juveniles (Table 4).

Based on the results of the logistic model, CDV seropositivity was significantly associated with age (p<0.0001), month (p=0.0002), and geographical location of sampling (p=0.0437) (Table 5). Although both age of the dog and geographical location had significant unadjusted association with the odds of *Ehrlichia spp.* seropositivity (Table 5) when both were offered to the model in a multivariable analysis, neither was significant. Therefore, there was no final multivariable model for *Ehrlichia spp.*

Controlling for the other two factors in the model, the odds of CDV seropositivity is 12 times higher among adult dogs than the juveniles (Table 6). Similarly, the odds of the dogs having seropositive results for CDV were 7.8 times higher in June than in July (Table 6). With respect to geographical location, only Khumaga (p=0.0014) and Shorobe (p=0.0481) had significantly different odds of canine seropositivity from MAWS with the odds of the dogs in Shorobe being 5.8 times higher than those of the reference group (MAWS) (Table 6). By contrast, the dogs in Khumaga had significantly lower odds (OR=0.072) of testing seropositive to CDV than dogs in MAWS. The odds of being seropositive for CDV among dogs from the other locations were not significantly different from that of MAWS. Based on the Hosmer-

Lemeshow Goodness-of-fit test, there is no evidence that the canine distemper model did not fit the data well (p=0.4813).

Table 3: Factor-specific seroprevalence of *Anaplasma spp.* and *D.immitis* among dogs in Botswana, 2015

Pathogen	n	Number of seropositive samples	Percentage of seropositive samples	P-value
Anaplasma spp.	**232**	**5**	**2.2**	
Sex				
Female	126	4	3.2	0.379
Male	106	1	0.9	
Age Category				
Adult	162	5	3.1	0.326
Juvenile	70	0	0	
Month				
June	104	1	1.0	0.383
July	128	4	3.1	
Location				
Boro	18	0	0	0.938
Etsha	32	0	0	
Khumaga	13	0	0	
MAWS	127	5	3.9	
Mathapane	16	0	0	
Sexaxa	8	0	0	
Shorobe	18	0	0	
D.immitis	**232**	**2**	**0.9**	
Sex				
Female	126	2	1.6	0.502
Male	106	0	0	
Age Category				
Adult	162	2	1.2	1.000
Juvenile	70	0	0	
Month				
June	104	1	1.0	1.000
July	128	1	0.8	
Location				
Boro	18	0	0	0.242
Etsha	32	0	0	
Khumaga	13	0	0	
MAWS	127	1	0.8	

Mathapane	16	0	0
Sexaxa	8	1	12.5
Shorobe	18	0	0

Table 4: Factor-specific seroprevalence of CAV and CPV among dogs in Botswana, 2015

Pathogen	n	Number of seropositive samples	Percentage of seropositive samples	P-value
**CAV**	**233**	**46**	**19.7**	
Sex				
Female	127	20	15.6	0.101
Male	106	26	24.5	
Age Category				
Adult	163	41	25.2	0.001
Juvenile	70	5	7.1	
Month				
June	104	26	25.0	0.097
July	129	20	15.5	
Location				
Boro	18	6	33.3	0.043
Etsha	32	5	15.6	
Khumaga	13	2	15.4	
MAWS	128	22	17.2	
Mathapane	16	8	50	
Sexaxa	8	0	0	
Shorobe	18	3	16.7	
**CPV**	**233**	**163**	**70.0**	
Sex				
Female	127	92	72.4	0.392
Male	106	71	67.0	
Age Category				
Adult	163	130	79.8	<0.001
Juvenile	70	33	47.1	
Month				
June	104	78	75.0	0.152
July	129	85	65.9	
Location				
Boro	18	15	83.3	0.025
Etsha	32	16	50	
Khumaga	13	6	46.2	
MAWS	128	92	71.9	
Mathapane	16	12	75	
Sexaxa	8	6	75	
Shorobe	18	16	88.9	

Table 5: Factor-specific seroprevalence of CDV and *Ehrlichia spp.* among dogs in Botswana, 2015

Pathogen	n	Number of seropositive samples	Percentage of seropositive samples	P-value
**CDV**	**233**	**109**	**46.8**	
Sex				
Female	127	62	48.8	0.512
Male	106	47	44.3	
Age Category				
Adult	163	99	60.7	<0.001
Juvenile	70	10	14.3	
Month				
June	104	71	68.3	<0.001
July	129	38	29.5	
Location				
Boro	18	14	77.8	<0.001
Etsha	32	6	18.8	
Khumaga	13	2	15.4	
MAWS	128	51	39.8	
Mathapane	16	13	81.3	
Sexaxa	8	6	75.0	
Shorobe	18	17	94.4	
Ehrlichia spp.	**233**	**49**	**21.0**	
Sex				
Female	126	28	22.1	0.748
Male	106	21	19.8	
Age Category				
Adult	163	40	24.5	0.054
Juvenile	70	9	12.9	
Month				
June	104	22	21.2	1.000
July	129	27	20.9	
Location				
Boro	18	3	16.7	<0.001
Etsha	32	0	0	
Khumaga	13	0	0	
MAWS	128	40	31.3	
Mathapane	16	3	18.8	
Sexaxa	8	2	25	
Shorobe	18	1	7.6	

Table 6: Results of multivariable logistic regression showing predictors of CDV sero-positivity among domestic dogs in Botswana, 2019

Predictor	Odds Ratio	95% Confidence Interval	P-value
**Age**			
Adult	12.4	4.8, 32.1	<0.0001
Juvenile	Referent	Referent	
**Month**			
June	7.8	2.6, 23.1	0.0002
July	Referent	Referent	
**Location**			
Boro	0.7	0.2, 3.3	0.8022
Etsha	0.7	0.2, 1.9	0.7233
Khumaga	0.07	0.01, 0.5	0.0014
Mathapane	1.2	0.2, 6.5	0.5889
Sexaxa	1.2	0.2, 9.6	0.6601
Shorobe	5.8	0.6, 57.8	0.0481
MAWS	Referent	Referent	

Discussion

Domestic animals can serve as disease reservoirs for wildlife. Wild animal populations, including various canid and felid species, have the potential to be infected by CDV, CPV, CAV, *D. immitis, B. burgdorferi, Ehrlichia spp.* and *Anaplasma spp.,* which are pathogens carried by the domestic animal population. Free-ranging cheetah (*Acinonyx jubatus*) in Namibia have been reported to have antibodies to CPV (though this test cross-reacts with feline panleukopenia virus) and CDV [15]. A captive breeding group of African wild dogs in Tanzania showed 94% mortality after infection with CDV [16], and another group of African wild dogs in Kenya had higher disease-related mortality rates from 21% to 50% and CDV antibodies rose from 1-4% to 76% over a three-year period [3]. In Chobe National Park, Botswana, in 1996, a pack of twelve African wild dogs was reduced to two animals following an outbreak of CDV [17]. While the prevalence in the current study was low (0.9%), *D. immitis* can infect wildlife, with reports of *D.*

immitis in a captive lion in Spain [18] and a captive black-footed cat in Florida [19]. Butler, et al. [20] indicated that domestic dogs in northwest Zimbabwe are a source of disease transmission for leopards (*Panthera pardus*), lions, and spotted hyenas (*Crocuta crocuta*), as these predator species feed on domestic dogs as prey. A survey of domestic dogs and African Wild dogs in Kenya from 2001 to 2009 showed 16% of African wild dogs and 48% of domestic dogs had been exposed to CDV, 25% of African wild dogs and 64% of domestic dogs had been exposed to CPV, and 80% of African wild dogs and 86% of domestic dogs had been exposed to *E. canis* [21]. Similar to the Kenya evaluation, the results of the present study revealed 46.8% CDV seropositititivity and 70.0% CPV seropositity in domestic dogs. However, we found lower seropositivity rates for *Ehrlichia spp.* (21.0%) compared to the Kenya study.

When comparing regional infectious disease prevalence differences, similar seroprevalence for viral diseases in domestic dogs was determined in Botswana as was reported in a similar study performed in Zimbabwe in 2012 [6]. A study of domestic dogs in northwest Zimbabwe reported that 34% had antibodies to CDV, 84% had antibodies to CPV, and 13% had antibodies for CAV [6]. These results are similar to those of the present study of 46.8% for CDV, 70.0% for CPV, and 19.7% for CAV. Another seroprevalence study evaluating domestic dogs in northeast Namibia in 1993 and 1994 found similar exposure to CDV (44.3%), but lower prevalence of CPV (47.1%) and higher prevalence of CAV (64.3%) [22]. Both Zimbabwe and Namibia border Botswana on its eastern and western edges, respectively. Viral diseases spread from animal to animal so localized differences in exposure are expected, but vectored pathogens depend on prevalence of the pathogen, prevalence of the vector, and on contact between the vector and the susceptible animal host. Williams, et al. [23] assessed prevalence of several hemoparasites, including *Ehrlichia spp.*, in domestic dogs, lions, spotted hyena, and African wild

dogs in 2009 to 2011 in Zambia, another country in southern Africa that borders Botswana, and samples were evaluated by polymerase chain reaction which only reveals active infections rather than current and past exposure. No carnivores had positive results for *E. canis* or *E. ewingii* [23], which does not rule out presence of ehrlichia in the study area.

As ticks are the vectors for *B. burgdorferi, Ehrlichia spp.* and *Anaplasma spp*, tick prevalence is a crucial factor in the spread of these pathogens. While no recent studies have reported tick prevalence in northern Botswana, Eygelaar, *et al.* [24] reported that African buffalo (*Syncerus caffer*) in the Okavango Delta had lower prevalence of tick-borne diseases than African buffalo in Chobe National Park, a region in northeast Botswana that is closer to the study region assessed by McRee, et al. [6]. Perhaps the same cause is responsible for the reduced tick-vectored diseases in African buffalo and domestic dogs in the Okavango Delta compared to northwest Zimbabwe. Eygelaar, et al. [24] hypothesized that veterinary fences in the Okavango Delta prevented direct contact between the African buffalo and cattle, which were not present in Chobe National Park. Perhaps these same fences reduce tick spread from wildlife to domestic dogs and vice versa, which limits the spread of the vector-borne diseases in the Okavango Delta which were not present in northwest Zimbabwe. More research must be performed to determine if the differences in *Ehrlichia spp*. seroprevalence is due to a reduction in total tick numbers or another cause, such as physical barriers. The high viral disease seroprevalence, similar to those in northwest Zimbabwe, is likely due to lack of vaccination. While strong efforts are being actively put forth by local not-for-profit organizations, the number of unvaccinated dogs remains much greater than the number of vaccinated dogs.

Factor-specific seroprevalence indicated significant associations between seropositivity to these common canine infectious diseases and age, month, and location. Adults are more likely

to be seropositive for CAV, CDV, and CPV, which is likely due to having more time to be exposed to the viruses than juveniles. There was a significant association between seropositivity for CDV and month with June having higher risk than July. Lastly, geographical location is a risk factor for CAV, CDV, and CPV because viral pathogens are transmitted either by direct contact or contact with bodily fluids. Therefore, geographical locations with high rates of these pathogens allow easy transmission to naïve individuals. Location was also a risk factor for *E. canis* perhaps because of tick concentrations in certain locations or due to an increase in the pathogen in the dogs of certain locations that perpetuates the elevated infection rate (ticks can spread the pathogen transstadially, but not transovarially [7]) .

While the viral pathogens evaluated in this study cannot infect humans, some of the vectored pathogens can affect humans. In addition to affecting domestic and wild animal populations, *E. canis* and *E. ewingii* have been reported in humans [25, 26]. *B. burgdorferi*, the causative organism for Lyme disease and *A. phagocytophilum* [27, 28] are also zoonotic. The 'One Health' paradigm, a collaborative approach to animal, human, and environmental health that recognizes their interconnectivity, is particularly important, since reducing disease risk in domestic animals will reduce disease risk in wildlife and human populations. By increasing vaccination and reducing tick burden in domestic dogs, human health and environmental health, in the case of wildlife, are improved [29, 30].

One of the limitations of this study was that serology detects exposure to the pathogen, but it does not determine the rate of active infections. Thus, sero-prevalence indicates that pathogen exposure has occurred, but current risk of infection is unknown. This aspect is important for human and wildlife health because new infections may increase disease burden in these populations.

Information regarding disease prevalence is necessary to determine domestic animal, wildlife, and human disease risk. This study reveals the need for local tick surveys to determine the cause of tick-borne pathogen prevalence differences between Botswana and surrounding countries. In conclusion, further disease testing and vaccination of both domestic dogs and wildlife would benefit domestic animals, wildlife, and humans in the Okavango Delta region of Botswana.

Acknowledgements

We would like to thank all of the staff at the Maun Animal Welfare Society, including Tana, Justine, KC, and Nation. We would also like to thank Hansje de Waard, Deirdre Halloran, and Anna Hewitt for generous assistance in sample collection.

References

1. Cleaveland S, Kaare M, Knobel D, Laurenson MK. Canine vaccination—providing broader benefits for disease control. Vet microbiol. 2006 Oct 5;117(1):43-50.
2. International Union for Conservation of Nature. The IUCN Red List of Threatened Species; 2019 [cited 2019 May 13]. Database [Internet]. Available from: http://www.iucnredlist.org/
3. Alexander KA, Appel MJ. African wild dogs (Lycaon pictus) endangered by a canine distemper epizootic among domestic dogs near the Masai Mara National Reserve, Kenya. J Wildl Dis. 1994 Oct;30(4):481-485.

4. Roelke-Parker ME, Munson L, Packer C, Kock R, Cleaveland S, Carpenter M, et al. A canine distemper virus epidemic in Serengeti lions (Panthera leo). Nature. 1996 Feb;379(6564):441.
5. Steinel A, Parrish CR, Bloom ME, Truyen U. Parvovirus infections in wild carnivores. J Wildl Dis. 2001 Jul;37(3):594-607.
6. McRee A, Wilkes RP, Dawson J, Parry R, Foggin C, Adams H, Kennedy MA. Serological detection of infection with canine distemper virus, canine parvovirus and canine adenovirus in communal dogs from Zimbabwe. J S Afr Vet Assoc. 2014 Jan;85(1):01-2.
7. Groves MG, Dennis GL, Amyx HL, Huxsoll DL. Transmission of Ehrlichia canis to dogs by ticks (Rhipicephalus sanguineus). Am J Vet Res. 1975 Jul;36(7):937-940.
8. Stuen S. Anaplasma phagocytophilum-the most widespread tick-borne infection in animals in Europe. Vet Res Commun. 2007 Aug 1;31(1):79-84.
9. Johnson RC, Schmid GP, Hyde FW, Steigerwalt AG, Brenner DJ. Borrelia burgdorferi sp. nov.: etiologic agent of Lyme disease. Int J Syst Evol Microbiol. 1984 Oct 1;34(4):496-497.
10. GRIEVE RB, LOK JB, GLICKMAN LT. Epidemiology of canine heartworm infection. Epidemiol Rev. 1983 Jan 1;5(1):220-246.
11. Harder TC, Osterhaus AD. Canine distemper virus—a morbillivirus in search of new hosts?. Trends Microbiol. 1997 Mar 1;5(3):120-124.
12. Goddard A, Leisewitz AL. Canine parvovirus. Vet Clin North Am Small Anim Pract. 2010 Nov 1;40(6):1041-1053.

13. Decaro N, Martella V, Buonavoglia C. Canine adenoviruses and herpesvirus. Vet Clin North Am Small Anim Pract. 2008 Jul 1;38(4):799-814.
14. Mmopelwa G, Blignaut JN. The Okavango Delta. The value of tourism: economic. South African Journal of Economic and Management Sciences. 2006 Mar 1;9(1):113-127.
15. Munson L, Marker L, Dubovi E, Spencer JA, Evermann JF, O'Brien SJ. Serosurvey of viral infections in free-ranging Namibian cheetahs (Acinonyx jubatus). J Wildl Dis. 2004 Jan;40(1):23-31.
16. van de Bildt MW, Kuiken T, Visee AM, Lema S, Fitzjohn TR, Osterhaus AD. Distemper outbreak and its effect on African wild dog conservation. Emerg Infect Dis. 2002 Feb;8(2):212.
17. Alexander KA, Kat PW, Munson LA, Kalake A, Appel MJ. Canine distemper-related mortality among wild dogs (Lycaon pictus) in Chobe National Park, Botswana. J Zoo Wildl Med. 1996 Sep;27(3):426-427
18. De Ybanez MR, Martínez-Carrasco C, Martínez JJ, Ortiz JM, Attout T, Bain O. Dirofilaria immitis in an African lion (Panthera leo). Vet Rec. 2006 Feb 18;158(7):24
19. Deem SL, Heard DJ, LaRock R. Heartworm (Dirofilaria immitis) disease and glomerulonephritis in a black-footed cat (Felis nigripes). J Zoo Wildl Med. 1998 Jun 1;29:199-202.
20. Butler JR, Du Toit JT, Bingham J. Free-ranging domestic dogs (Canis familiaris) as predators and prey in rural Zimbabwe: threats of competition and disease to large wild carnivores. Biol Conserv. 2004 Feb 1;115(3):369-378.

21. Woodroffe R, Prager KC, Munson L, Conrad PA, Dubovi EJ, Mazet JA. Contact with domestic dogs increases pathogen exposure in endangered African wild dogs (Lycaon pictus). PLoS One. 2012 Jan 6;7(1):e30099.
22. Laurenson K, Van Heerden J, Stander P, Van Vuuren MJ. Seroepidemiological survey of sympatric domestic and wild dogs (Lycaon pictus) in Tsumkwe District, northeastern Namibia. Onderstepoort J Vet Res. 1997 Dec 1;64:313-316.
23. Williams BM, Berentsen A, Shock BC, Teixiera M, Dunbar MR, Becker MS, et al. Prevalence and diversity of Babesia, Hepatozoon, Ehrlichia, and Bartonella in wild and domestic carnivores from Zambia, Africa. Parasitol Res. 2014 Mar 1;113(3):911-918.
24. Eygelaar D, Jori F, Mokopasetso M, Sibeko KP, Collins NE, Vorster I, et al. Tick-borne haemoparasites in African buffalo (Syncerus caffer) from two wildlife areas in Northern Botswana. Parasit Vectors. 2015 Dec;8(1):26.
25. Conrad ME. Ehrlichia canis: a tick-borne rickettsial-like infection in humans living in the southeastern United States. Am J Med Sci. 1989 Jan 1;297(1):35-37.
26. Buller RS, Arens M, Hmiel SP, Paddock CD, Sumner JW, Rikihisa Y, et al. Ehrlichia ewingii, a newly recognized agent of human ehrlichiosis. N Engl J Med. 1999 Jul 15;341(3):148-155.
27. Murphree Bacon R, Kugeler KJ, Mead PS. Surveillance for Lyme disease--United States, 1992-2006. 2008.
28. Dumler JS, Choi KS, Garcia-Garcia JC, Barat NS, Scorpio DG, Garyu JW, et al. Human granulocytic anaplasmosis and Anaplasma phagocytophilum. Emerg Infect Dis. 2005 Dec;11(12):1828.

29. Day MJ. One health: the importance of companion animal vector-borne diseases. Parasit Vectors. 2011 Dec;4(1):49.

30. Dantas-Torres F, Chomel BB, Otranto D. Ticks and tick-borne diseases: a One Health perspective. Trends Parasitol. 2012 Oct 1;28(10):437-446.

Manuscript Click here to access/download;Manuscript;Manuscript.docx

Seroprevalence of viral and vector-borne pathogens in domestic dogs (*Canis familiaris)* in northern Botswana

Short title: Prevalence of antibodies to infectious agents in Botswanan dogs

Riley E. Thompson[1], Hayley Adams[2], Agricola Odoi[3], Melissa A. Kennedy[3]

[1]Department of Large Animal Clinical Sciences, College of Veterinary Medicine, University of Tennessee, Knoxville, TN, USA, [2]Silent Heroes Foundation, USA, [3]Department of Biomedical and Diagnostic Sciences, College of Veterinary Medicine, University of Tennessee, Knoxville, TN, USA

Correspondence to:

Melissa Kennedy

Email:

mkenned2@utk.edu

Author contributions

R.E.T. (University of Tennessee) collected samples, performed ELISA testing, and was involved in manuscript writing and edits, H.A. (Silent Heroes Foundation) designed experiments and arranged local logistics, A.O. (University of Tennessee) performed statistical analysis and was involved in manuscript writing and edits, and M.A.K. (University of Tennessee) was the project leader and was responsible for experimental design and data analysis.

Abstract

Background: Domestic dogs (*Canis familiaris*) have the potential to act as disease reservoirs for wildlife and are important sentinels for common circulating pathogens. Therefore, the infectious agent seroprevalence among domestic dogs in northern Botswana may be indicative of pathogen exposure of various wildlife species. The objective of this study was to assess the seroprevalence of *Ehrlichia* spp., *Borrelia burgdorferi*, *Anaplasma* spp., *Dirofilaria immitis*, canine adenovirus, canine parvovirus-2, and canine distemper virus in domestic dogs as proxies of disease prevalence in the local wildlife in the Okavango Delta region of Botswana. Statistical analysis assessed crude and factor-specific seroprevalence proportions and assessed the factors age, sex, and geographical location as predictors of seropositivity. Logistic regression was used to identify adjusted predictors of seropositivity for each of the pathogens of interest.

Results: Samples from 233 dogs in 7 locations in Maun, Botswana, and surrounding villages were collected and serologically analyzed. No dogs were seropositive for *B. burgdorferi,* while low seroprevalence proportions were observed for *Anaplasma* spp. (2.2%) and *D. immitis* (0.9%). Higher seroprevalence proportions were observed for the tick-borne pathogen, *Ehrlichia* spp. (21.0%), and 19.7% were seropositive for canine adenovirus (hepatitis). The highest seroprevalence proportions were for canine parvovirus-2 (70.0%) and canine distemper virus (44.8%). The predictors of seropositivity revealed that adults were more likely to be seropositive for canine adenovirus, canine distemper virus, and canine parvovirus-2 than juveniles, and location was a risk factor for canine adenovirus, canine distemper virus, canine parvovirus-2, and *Ehrlichia* spp.

Conclusions: Results indicate a need for tick prevalence data and that increasing vaccination campaigns for domestic dogs may improve the health of domestic animals, and potentially wildlife and humans in the Okavango Delta since viral and vector-borne bacterial pathogens can be transmitted between them.

Introduction

Vaccination of domestic dogs has been reported as a method of wildlife conservation [1] with the implication that prevalence of transmissible diseases in the domestic canine population has the potential to affect disease burden in wildlife, including both wild felids and wild canids. African wild dogs (*Lycaon pictus*), a wild canid species in sub-Saharan Africa, are endangered according to the International Union for the Conservation of Nature Redlist [2], and the black-footed cat (*Felis nigripes*), cheetah (*Acinonyx jubatus*), and lion (*Panthera leo*) are all vulnerable species in Botswana [2] that can be negatively impacted by domestic dog viral and vector-borne bacterial pathogens [3-5]. Capturing a sufficient number of these wild canids and felids to perform seroprevalence surveys is not always feasible due to the risk associated with anesthesia necessary to collect blood samples from these animals. This is particularly concerning due to the low numbers of individuals as indicated by their conservation status. McRee, et al. [6] performed a seroprevalence study of viral pathogens in domestic dogs in northwest Zimbabwe as a representation of wildlife viral disease agent prevalence in the region, particularly in African wild dogs.

Ehrlichia spp., *Anaplasma* spp., and *Borrelia burgdorferi* (Lyme disease) are bacterial pathogens that are transmitted by tick bites [7-9]. *Dirofilaria immitis*, or heartworm disease, is a blood-borne parasite transmitted by mosquito bites [10], and canine distemper virus (CDV),

parvovirus-2 (CPV2), and adenovirus (CAV) are viral diseases transmitted between individuals [11-13]. All of these common pathogens in dogs can cause serious illness and/or death. While the viral diseases, Lyme disease, and heartworm disease can be prevented either by vaccination or monthly heartworm preventative medication, many communities in southern Africa do not have the resources to pay for these medications for their animals. Thus, these preventable diseases may be widespread.

Botswana is a land-locked country in southern Africa and is home to the Okavango Delta, a diverse wetland habitat. Not only is the Okavango Delta home to countless species, it is the center for tourism in the country, which has become the second most important industry in Botswana after diamond mining [14]. The Okavango Delta of Botswana is rich with wildlife which have the chance to interact with domestic animal populations. This potentially results in cross-transmission of infections between domestic and wild animals implying that infectious disease exposure in domestic animals might mirror those of wildlife. As disease prevalence of common infectious diseases in wild carnivores is unknown in the Okavango Delta, this presents the opportunity to use domestic dogs as sentinels for infectious disease exposure in wildlife. Therefore, the objective of this study was to assess the seroprevalence of common infectious disease agents (*Ehrlichia* spp., *Borrelia burgdorferi*, *Anaplasma* spp., *Dirofilaria immitis*, CDV, CAV, and CPV2) in domestic dogs in Maun, Botswana, an area adjacent to the Okavango Delta, as a proxy for seroprevalence that would be expected in the wild canid and felid populations. Our hypothesis is that these agents will be present at significant levels in domestic dogs indicating their presence in this geographic locale.

Materials and Methods

Animals

The majority (n=128/233) of blood samples from domestic dogs were collected using a convenience sampling strategy at the Maun Animal Welfare Society (MAWS) in Maun, Botswana, from dogs who were presented for castration and vaccination and under the direct supervision of the staff veterinarian. The remaining blood samples (n=105/233) were collected in surrounding villages also under veterinary supervision. Informed consent was obtained verbally from each owner of dogs sampled. The uncastrated domestic dogs in this area were highly unlikely to have received any vaccines except rabies vaccination and be seropositive to the viral diseases due to vaccination cross-reaction. This is because MAWS is the main veterinary clinic in the area for the low income population, and they will not vaccinate animals unless they are also castrated at the time of vaccination. Therefore, animals that were reproductively intact are likely to be unvaccinated. Demographic data was recorded at the time of sample collection including sex, breed, and approximate age of 'juvenile' or 'adult' based on dentition and overall appearance. Blood samples of 1-2ml volume were collected from a peripheral vein, transported on ice, and whole blood stored at 4°C until testing within 48 hours.

Sample Analyses

One to two ml of whole blood were collected in an EDTA tube from 233 dogs. Seroprevalence for CAV-, CPV2-, and CDV-specific antibodies were assessed using Biogal ImmunoComb Canine VacciCheck (Biogal Galed Laboratories, Kibbutz Galed, Israel) per manufacturer instructions. The Biogal Titer Check results were reported with a change of color and 'negative' (no color development), 'positive' (equal color development to the reference spot- 1:16 VN for CAV, 1:80 HI for CPV2, and 1:32 VN for CDV), 'highly positive' (color development greater

than the reference spot), or 'inconclusive' (very faint color development) as the range of possible results. The vector-borne diseases, *Ehrlichia* spp., *Borrelia burgdorferi*, *Anaplasma* spp., and *Dirofilaria immitis*, were assessed with IDEXX 4DX SNAP ELISA (IDEXX, Westbrook, Maine, USA) per manufacturer instructions. The IDEXX SNAP ELISA results were reported as either 'positive' or 'negative' by a color change. Rabies seroprevalence was not assessed.

Statistical Analysis

All statistical analyses were performed in SAS (Statistical Analysis Systems). Crude and factor-specific seroprevalence proportions of *Ehrlichia* spp., *Borrelia burgdorferi*, *Anaplasma* spp., *Dirofilaria immitis*, CDV, CAV and CPV as well as their 95% exact confidence intervals were computed. The factors considered were age, sex, and location. Associations between seroprevalence and each of the above factors were assessed using the Chi-square or Fishers Exact tests as appropriate. Significance was set at $\alpha = 0.05$ for all statistical tests. Logistic regression was used to identify adjusted predictors of seropositivity for each of the pathogens of interest.

Results

Animal demographics

A total of 233 dogs were tested (Table 1). However, at least one of the dogs had missing data and so some of the statistical analyses were performed on only 232 records. Females dogs made up the majority (54.5%) of the sampled dogs. Dogs were sampled during the months of June and July 2015 with the majority (55.4%) of the dogs being sampled in July. The age group distribution was 69.7% adults and 30.0% juveniles. Whole blood samples were collected at

seven locations in Maun, Botswana, and surrounding villages: MAWS (n=128), Khumaga (n=13), Boro (n=18), Mathapane (n=16), Shorobe (n=18), Sexaxa (n=8), and Etsha (n=32). All dogs were of mixed breed. Rabies seroprevalence studies were not assessed as vaccination status was unknown, and no point-of-care ELISA or similar was available. The latter would have been necessary since canine serum could not be transported for testing.

Table 1: Characteristics of dogs included in a seroprevalence assessment of prior exposure to common pathogens in Botswana, 2015

Variable (n=233)	Number	Percent	95% Exact Binomial Confidence Interval
Sex			
Female	127	54.5	47.9, 61.0
Male	106	45.5	3.90, 52.1
Age Category			
Adult	163	69.7	63.6, 75.8
Juvenile	70	30.0	24.2, 36.3
Location			
Boro	18	7.7	4.6, 11.9
Etsha	32	13.7	9.6, 18.8
Khumaga	13	5.6	3.0, 9.4
MAWS	128	54.9	48.3, 61.4
Mathapane	16	6.9	4.0, 10.9
Sexaxa	8	3.4	1.5, 6.7
Shorobe	18	7.7	4.6, 11.9
Month			
June 2015	104	44.6	38.1, 51.0
July 2015	129	55.4	48.7, 61.8

Crude seroprevalence

Of 232 dogs tested for *Borrelia burgdorferi*, *Anaplasma* spp and *Dirofilaria*, 0% were seropositive for *Borrelia burgdorferi*, 2.2% were seropositive for *Anaplasma spp.*, and 0.9% were seropositive for *Dirofilaria immitis* (Table 2). One dog had missing data for the above 3 pathogens. Out of the 233 dogs, 21.0% were seropositive for *Ehrlichia spp.*, 19.7% were seropositive for CAV, 70% were seropositive for CPV2, and 46.8% were seropositive for CDV.

Table 2: Crude seroprevalence of selected pathogens among domestic dogs in Botswana, 2015

Pathogen	Number of seropositive samples	Percentage of seropositive samples	95% CI1
Anaplasma spp.(n=232)*	5	2.2	0.7, 5.0
B.burgdorferi (n=232)*	0	0	0, 1.6
D.immitis (n=232)*	2	0.9	0.1, 3.1
Distemper Virus (n=233)	109	46.8	40.2, 53.4
Ehrlichia spp. (n=233)	49	21.0	16.0, 26.8
Adenovirus (n=233)	46	19.7	14.8, 24.4
Parvovirus (n=233)	163	70.0	63.6, 75.8

* There was missing data for the pathogens *Anaplasma*, *B.burgdorferi*, and *D.immitis* and so the sample size was 232 and not 233

95% CI: 95% confidence interval

Predictors of seropositivity

Based on univariable analyses, none of the variables assessed (sex, age, month, or location) had a statistically significant (p>0.05) association with seropositivity of either *Anaplasma* spp. or *D. immitus* (Table 3a). Although both age of the dog and geographical location had significant unadjusted association with *Ehrlichia* spp. seropositivity (Table 3b), when both were offered to the model in a multivariable analysis, only age was significant (p=0.0846) (Table 4).

Both age of the dog and location had significant unadjusted associations with the odds of CAV seropositivity, only age (OR=4.4; p<0.0003) was significant in the final; implying that the odds of CAV seropositivity was 4.4 times higher in adult dogs than in juveniles (Table 4). Similarly, only age had a significant association in the final model for CPV with adults also having 4.4 times higher odds of CPV2 seropositivity (OR=4.4; p<0.0001) than juveniles (Table 4).

For CDV, the final logistic model showed that CDV seropositivity was significantly associated with age (p<0.0001), month (p=0.0002), and geographical location of sampling

(p=0.0437) (Table 4). Controlling for the other two factors in the model, the odds of CDV seropositivity was 12 times higher among adult dogs than juveniles (Table 4). Similarly, the odds of the dogs having seropositive results for CDV were 7.8 times higher in June than in July (Table 4). With respect to geographical location, only Khumaga (p=0.0014) and Shorobe (p=0.0481) had significantly different odds of canine seropositivity from MAWS with the odds of seropositivity for CDV among dogs in Shorobe being 5.8 times higher than those of the reference group (MAWS) (Table 4). By contrast, the dogs in Khumaga had significantly lower odds (OR=0.07) of testing seropositive to CDV than dogs in MAWS. The odds of being seropositive for CDV among dogs from the other locations were not significantly different from that of MAWS. Based on the Hosmer-Lemeshow Goodness-of-fit test, there is no evidence that the canine distemper model did not fit the data well (p=0.4813).

Table 3a: Factor-specific seroprevalence of selected pathogens among dogs in Botswana, 2015

	Anaplasma spp. (n=232)				***D.immitis (n=232)***				**Adenovirus (n=233)**				**Parvovirus (n=233)**			
**Pathogen**	**n[2]**	**# pos[1]**	**% pos[1]**	**p[3]**	**n[2]**	**# pos[1]**	**% pos[1]**	**p[3]**	**n[2]**	**# pos[1]**	**% pos[1]**	**p[3]**	**n[2]**	**# pos[1]**	**% pos[1]**	**p[3]**
Total	232	5	2.2		232	2	0.9		233	46	19.7		233	163	70.0	
Sex				0.379				0.502								
Female	126	4	3.2		126	2	1.6		127	20	15.6	0.101	127	92	72.4	0.392
Male	106	1	0.9		106	0	0		106	26	24.5		106	71	67.0	
Age Category				0.326				1.000								
Adult	162	5	3.1		162	2	1.2		163	41	25.2	0.001	163	130	79.8	<0.001
Juvenile	70	0	0		70	0	0		70	5	7.1		70	33	47.1	
Month				0.383				1.000								
June	104	1	1.0		104	1	1.0		104	26	25.0	0.097	104	78	75.0	0.152
July	128	4	3.1		128	1	0.8		129	20	15.5		129	85	65.9	
Location				0.938				0.242								
Boro	18	0	0		18	0	0		18	6	33.3	0.043	18	15	83.3	0.025
Etsha	32	0	0		32	0	0		32	5	15.6		32	16	50	
Khumaga	13	0	0		13	0	0		13	2	15.4		13	6	46.2	
MAWS	127	5	3.9		127	1	0.8		128	22	17.2		128	92	71.9	
Mathapane	16	0	0		16	0	0		16	8	50		16	12	75	
Sexaxa	8	0	0		8	1	12.5		8	0	0		8	6	75	
Shorobe	18	0	0		18	0	0		18	3	16.7		18	16	88.9	

[1]Positive

[2]Number of samples tested (note that there is missing data for *Anaplasma* and *D.immitis* and so there total samples is 232 instead of 233)

[3]p-value

Table 3b: Factor-specific seroprevalence of selected pathogens among dogs in Botswana, 2015

	Distemper spp. (n=233)				*Ehrlichia (n=233)*			
**Pathogen**	**n[2]**	**# pos[1]**	**% pos[1]**	**p[3]**	**n[2]**	**# pos[1]**	**% pos[1]**	**p[3]**
Total	233	109	46.8		233	49	21.0	
Sex								
Female	127	62	48.8	0.512	126	28	22.1	0.748
Male	106	47	44.3		106	21	19.8	
Age Category								
Adult	163	99	60.7	<0.001	163	40	24.5	0.054
Juvenile	70	10	14.3		70	9	12.9	
Month								
June	104	71	68.3	<0.001	104	22	21.2	1.000
July	129	38	29.5		129	27	20.9	
Location								
Boro	18	14	77.8	<0.001	18	3	16.7	<0.001
Etsha	32	6	18.8		32	0	0	
Khumaga	13	2	15.4		13	0	0	
MAWS	128	51	39.8		128	40	31.3	
Mathapane	16	13	81.3		16	3	18.8	
Sexaxa	8	6	75.0		8	2	25	
Shorobe	18	17	94.4		18	1	7.6	

[1]Positive
[2]Number of samples tested (note that there is missing data for *Anaplasma* and *D.immitis* and so there total samples is 232 instead of 233)
[3]p-value

Table 4: Results of multivariable logistic regression showing predictors of CDV sero-positivity among domestic dogs in Botswana, 2019

	Canine Adenovirus Model			Canine Parvovirus Model			Canine Distemper Virus Model			Erhlichia canis Model		
**Predictor**	**OR[1]**	**95% CI[2]**	**P-value**	**OR[1]**	**95% CI[2]**	**P-value**	**OR[1]**	**95% CI[2]**	**P-value**	**OR[1]**	**95% CI[2]**	**P-value**
**Age**			**0.0031**			**<0.0001**			**<0.0001**			**0.0486**
Adult	4.4	1.6,11.6	0.0031	4.4	2.4, 8.1	<0.0001	12.4	4.8, 32.1	<0.0001	2.2	1.0, 4.8	0.0486
Juvenile	Ref[3]	Ref[3]		Ref[3]	Ref[3]		Ref[3]	Ref[3]				
**Month**	NS[4]			NS[4]					**0.0002**	NS[4]		
June							7.8	2.6, 23.1	0.0002			
July							Ref[3]	Ref[3]				
**Location**	NS[4]			NS[4]					**0.0437**	NS[4]		
Boro							0.7	0.2, 3.3	0.8022			
Etsha							0.7	0.2, 1.9	0.7233			
Khumaga							0.07	0.01, 0.5	0.0014			
Mathapane							1.2	0.2, 6.5	0.5889			
Sexaxa							1.2	0.2, 9.6	0.6601			
Shorobe							5.8	0.6, 57.8	0.0481			
MAWS							Ref[3]	Ref[3]				

[1]Odds Ratio
[2]95% Confidence Interval
[3]Reference Group
[4]Not statistically significant (p>0.05) and therefore not included in the final model

Discussion

Domestic animals can serve as disease reservoirs for wildlife. Wild animal populations, including various canid and felid species, have the potential to be infected by CDV, CPV2, CAV, *D. immitis, B. burgdorferi, Ehrlichia* spp. and *Anaplasma* spp., which are pathogens carried by the domestic animal population. Free-ranging cheetah (*Acinonyx jubatus*) in Namibia have been reported to have antibodies to CPV2 (though this test cross-reacts with feline panleukopenia virus) and CDV [15]. A captive breeding group of African wild dogs in Tanzania showed 94% mortality after infection with CDV [16], and another group of African wild dogs in Kenya had higher disease-related mortality rates from 21% to 50% and CDV antibodies rose from 1-4% to 76% over a three-year period [3]. In Chobe National Park, Botswana, in 1996, a pack of twelve African wild dogs was reduced to two animals following an outbreak of CDV [17]. While the prevalence in the current study was low (0.9%), *D. immitis* can infect wildlife, with reports of *D. immitis* in a captive lion in Spain [18] and a captive black-footed cat in Florida [19]. Butler, et al. [20] indicated that domestic dogs in northwest Zimbabwe are a source of disease transmission for leopards (*Panthera pardus*), lions, and spotted hyenas (*Crocuta crocuta*), as these predator species feed on domestic dogs as prey. A survey in Kenya from 2001 to 2009 showed 16%, 25%, and 80% of African wild dogs and 48%, 64% and 86% of domestic dogs had been exposed to CDV, CPV2, and *E. canis* respectively [21]. Similar to the Kenya evaluation, the results of the present study revealed 46.8% CDV seroposititivity and 70.0% CPV2 seropositivy in domestic dogs. However, we found lower seropositivity rates for *Ehrlichia* spp. (21.0%) compared to the Kenya study.

When comparing regional infectious disease prevalence differences, similar seroprevalence for viral diseases in domestic dogs was determined in Botswana as was reported in a similar study performed in Zimbabwe in 2012 [6]. A study of domestic dogs in northwest Zimbabwe reported that 34% had antibodies to CDV, 84% had antibodies to CPV2, and 13% had antibodies for CAV [6]. These results are similar to those of the present study of 46.8% for CDV, 70.0% for CPV2, and 19.7% for CAV. Another seroprevalence study evaluating domestic dogs in northeast Namibia in 1993 and 1994 found similar exposure to CDV (44.3%), but lower prevalence of CPV2 (47.1%) and higher prevalence of CAV (64.3%) [22]. Both Zimbabwe and Namibia border Botswana on its eastern and western edges, respectively. Viral diseases spread from animal to animal so localized differences in exposure are expected, but vectored pathogens depend on prevalence of the pathogen, prevalence of the vector, and on contact between the vector and the susceptible animal host. Williams, et al. [23] assessed prevalence of several hemoparasites, including *Ehrlichia* spp., in domestic dogs, lions, spotted hyena, and African wild dogs in 2009 to 2011 in Zambia, another country in southern Africa that borders Botswana, and samples were evaluated by polymerase chain reaction which only reveals active infections rather than current and past exposure. No carnivores had positive results for *E. canis* or *E. ewingii* [23], which does not rule out presence of ehrlichia in the study area.

As ticks are the vectors for *B. burgdorferi*, *Ehrlichia spp.* and *Anaplasma spp*, tick prevalence is a crucial factor in the spread of these pathogens. *Ixodes* spp. are the vectors for *B. burgdorferi*, and *Rhipicephalus* spp. are the primary vectors for *E. canis* and *Anaplasma* spp., although other tick species have been reported to transmit both pathogens. While no recent studies have reported tick prevalence in northern Botswana, Eygelaar, *et al.* [24] reported that African buffalo (*Syncerus caffer*) in the

Okavango Delta had lower prevalence of tick-borne diseases than African buffalo in Chobe National Park, a region in northeast Botswana that is closer to the study region assessed by McRee, et al. [6]. Perhaps the same cause is responsible for the reduced tick-vectored diseases in African buffalo and domestic dogs in the Okavango Delta compared to northwest Zimbabwe. Eygelaar, et al. [24] hypothesized that veterinary fences in the Okavango Delta prevented direct contact between the African buffalo and cattle, which were not present in Chobe National Park. Perhaps these same fences reduce tick spread from wildlife to domestic dogs and vice versa, which limits the spread of the vector-borne diseases in the Okavango Delta which were not present in northwest Zimbabwe. More research must be performed to determine if the differences in *Ehrlichia* spp. seroprevalence is due to a reduction in total tick numbers or another cause, such as physical barriers. The high viral disease seroprevalence, similar to those in northwest Zimbabwe, is likely due to lack of vaccination. While strong efforts are being actively put forth by local not-for-profit organizations, the number of unvaccinated dogs remains much greater than the number of vaccinated dogs.

Factor-specific seroprevalence indicated significant associations between seropositivity to these common canine infectious diseases and age, month, and location. Adults are more likely to be seropositive for CAV, CDV, and CPV2, which is likely due to having more time to be exposed to the viruses than juveniles. There was a significant association between seropositivity for CDV and month with June having higher risk than July. The reason for this is unclear and will require further investigation in future studies. Lastly, geographical location is a risk factor for CAV, CDV, and CPV2 because viral pathogens are transmitted either by direct contact or contact with bodily fluids. Therefore, geographical locations with high rates of these pathogens allow easy transmission to naïve individuals. Location was also a risk factor for *E. canis* perhaps because of tick concentrations in certain

locations or due to an increase in the pathogen in the dogs of certain locations that perpetuates the elevated infection rate (ticks can spread the pathogen transtadially, but not transovarially [7]) .

While the viral pathogens evaluated in this study cannot infect humans, some of the vectored pathogens can affect humans. In addition to affecting domestic and wild animal populations, *E. canis* and *E. ewingii* have been reported in humans [25, 26]. *B. burgdorferi*, the causative organism for Lyme disease, and *A. phagocytophilum* [27, 28] are also zoonotic. The 'One Health' paradigm, a collaborative approach to animal, human, and environmental health that recognizes their interconnectivity, is particularly important, since reducing disease risk in domestic animals will reduce disease risk in wildlife and human populations. By increasing vaccination in domestic dogs, human health and environmental health, in the case of wildlife, are improved [29, 30].

One of the limitations of this study was that serology detects exposure to the pathogen, but it does not determine the rate of active infections. Thus, sero-prevalence indicates that pathogen exposure has occurred, but current risk of infection is unknown. This aspect is important for human and wildlife health because new infections may increase disease burden in these populations.

Conclusions

Information regarding disease prevalence is necessary to determine domestic animal, wildlife, and human disease risk. This study reveals the need for local tick surveys to determine the cause of tick-borne pathogen prevalence differences between Botswana and surrounding countries. In conclusion, further disease testing and vaccination of both domestic dogs and wildlife would benefit domestic animals, wildlife, and humans in the Okavango Delta region of Botswana.

Acknowledgements

We would like to thank all of the staff at the Maun Animal Welfare Society, including Tana, Justine, KC, and Nation. We would also like to thank Hansje de Waard, Deirdre Halloran, and Anna Hewitt for generous assistance in sample collection.

References

1. Cleaveland S, Kaare M, Knobel D, Laurenson MK. Canine vaccination—providing broader benefits for disease control. Vet microbiol. 2006 Oct 5;117(1):43-50.
2. International Union for Conservation of Nature. The IUCN Red List of Threatened Species; 2019 [cited 2019 May 13]. Database [Internet]. Available from: http://www.iucnredlist.org/
3. Alexander KA, Appel MJ. African wild dogs (Lycaon pictus) endangered by a canine distemper epizootic among domestic dogs near the Masai Mara National Reserve, Kenya. J Wildl Dis. 1994 Oct;30(4):481-485.
4. Roelke-Parker ME, Munson L, Packer C, Kock R, Cleaveland S, Carpenter M, et al. A canine distemper virus epidemic in Serengeti lions (Panthera leo). Nature. 1996 Feb;379(6564):441.
5. Steinel A, Parrish CR, Bloom ME, Truyen U. Parvovirus infections in wild carnivores. J Wildl Dis. 2001 Jul;37(3):594-607.

6. McRee A, Wilkes RP, Dawson J, Parry R, Foggin C, Adams H, Kennedy MA. Serological detection of infection with canine distemper virus, canine parvovirus and canine adenovirus in communal dogs from Zimbabwe. J S Afr Vet Assoc. 2014 Jan;85(1):01-2.
7. Groves MG, Dennis GL, Amyx HL, Huxsoll DL. Transmission of *Ehrlichia canis* to dogs by ticks (*Rhipicephalus sanguineus*). Am J Vet Res. 1975 Jul;36(7):937-940.
8. Stuen S. *Anaplasma phagocytophilum*-the most widespread tick-borne infection in animals in Europe. Vet Res Commun. 2007 Aug 1;31(1):79-84.
9. Johnson RC, Schmid GP, Hyde FW, Steigerwalt AG, Brenner DJ. *Borrelia burgdorferi* sp. nov.: etiologic agent of Lyme disease. Int J Syst Evol Microbiol. 1984 Oct 1;34(4):496-497.
10. Grieve RB, Lok JB, Glickman LT. Epidemiology of canine heartworm infection. Epidemiol Rev. 1983 Jan 1;5(1):220-246.
11. Harder TC, Osterhaus AD. Canine distemper virus—a morbillivirus in search of new hosts?. Trends Microbiol. 1997 Mar 1;5(3):120-124.
12. Goddard A, Leisewitz AL. Canine parvovirus. Vet Clin North Am Small Anim Pract. 2010 Nov 1;40(6):1041-1053.
13. Decaro N, Martella V, Buonavoglia C. Canine adenoviruses and herpesvirus. Vet Clin North Am Small Anim Pract. 2008 Jul 1;38(4):799-814.
14. Mmopelwa G, Blignaut JN. The Okavango Delta. The value of tourism: economic. South African Journal of Economic and Management Sciences. 2006 Mar 1;9(1):113-127.

15. Munson L, Marker L, Dubovi E, Spencer JA, Evermann JF, O'Brien SJ. Serosurvey of viral infections in free-ranging Namibian cheetahs (Acinonyx jubatus). J Wildl Dis. 2004 Jan;40(1):23-31.
16. van de Bildt MW, Kuiken T, Visee AM, Lema S, Fitzjohn TR, Osterhaus AD. Distemper outbreak and its effect on African wild dog conservation. Emerg Infect Dis. 2002 Feb;8(2):212.
17. Alexander KA, Kat PW, Munson LA, Kalake A, Appel MJ. Canine distemper-related mortality among wild dogs (Lycaon pictus) in Chobe National Park, Botswana. J Zoo Wildl Med. 1996 Sep;27(3):426-427
18. De Ybanez MR, Martínez-Carrasco C, Martínez JJ, Ortiz JM, Attout T, Bain O. *Dirofilaria immitis* in an African lion (Panthera leo). Vet Rec. 2006 Feb 18;158(7):24
19. Deem SL, Heard DJ, LaRock R. Heartworm (*Dirofilaria immitis*) disease and glomerulonephritis in a black-footed cat (*Felis nigripes*). J Zoo Wildl Med. 1998 Jun 1;29:199-202.
20. Butler JR, Du Toit JT, Bingham J. Free-ranging domestic dogs (*Canis familiaris*) as predators and prey in rural Zimbabwe: threats of competition and disease to large wild carnivores. Biol Conserv. 2004 Feb 1;115(3):369-378.
21. Woodroffe R, Prager KC, Munson L, Conrad PA, Dubovi EJ, Mazet JA. Contact with domestic dogs increases pathogen exposure in endangered African wild dogs (*Lycaon pictus*). PLoS One. 2012 Jan 6;7(1):e30099.
22. Laurenson K, Van Heerden J, Stander P, Van Vuuren MJ. Seroepidemiological survey of sympatric domestic and wild dogs (*Lycaon pictus*) in Tsumkwe District, northeastern Namibia. Onderstepoort J Vet Res. 1997 Dec 1;64:313-316.

23. Williams BM, Berentsen A, Shock BC, Teixiera M, Dunbar MR, Becker MS, et al. Prevalence and diversity of *Babesia*, *Hepatozoon*, *Ehrlichia*, and *Bartonella* in wild and domestic carnivores from Zambia, Africa. Parasitol Res. 2014 Mar 1;113(3):911-918.

24. Eygelaar D, Jori F, Mokopasetso M, Sibeko KP, Collins NE, Vorster I, et al. Tick-borne haemoparasites in African buffalo (*Syncerus caffer*) from two wildlife areas in Northern Botswana. Parasit Vectors. 2015 Dec;8(1):26.

25. Conrad ME. *Ehrlichia canis*: a tick-borne rickettsial-like infection in humans living in the southeastern United States. Am J Med Sci. 1989 Jan 1;297(1):35-37.

26. Buller RS, Arens M, Hmiel SP, Paddock CD, Sumner JW, Rikihisa Y, et al. *Ehrlichia ewingii*, a newly recognized agent of human ehrlichiosis. N Engl J Med. 1999 Jul 15;341(3):148-155.

27. Murphree Bacon R, Kugeler KJ, Mead PS. Surveillance for Lyme disease--United States, 1992-2006. 2008.

28. Dumler JS, Choi KS, Garcia-Garcia JC, Barat NS, Scorpio DG, Garyu JW, et al. Human granulocytic anaplasmosis and *Anaplasma phagocytophilum*. Emerg Infect Dis. 2005 Dec;11(12):1828.

29. Day MJ. One health: the importance of companion animal vector-borne diseases. Parasit Vectors. 2011 Dec;4(1):49.

30. Dantas-Torres F, Chomel BB, Otranto D. Ticks and tick-borne diseases: a One Health perspective. Trends Parasitol. 2012 Oct 1;28(10):437-446.

Manuscript

Click here to access/download;Manuscript;Botswana manuscript PLOS submission (2).docx

Seroprevalence of viral and vector-borne bacterial pathogens in domestic dogs (*Canis familiaris)* in northern Botswana

Short title: Infectious disease prevalence in dogs in Botswana

Riley E. Thompson[1], Hayley Adams[2], Agricola Odoi[3], Melissa A. Kennedy[3]

[1]Department of Large Animal Clinical Sciences, College of Veterinary Medicine, University of Tennessee, Knoxville, TN, USA, [2]Silent Heroes Foundation, USA, [3]Department of Biomedical and Diagnostic Sciences, College of Veterinary Medicine, University of Tennessee, Knoxville, TN, USA

Correspondence to:

Melissa Kennedy

Email:

mkenned2@utk.edu

Author contributions

R.E.T. (University of Tennessee) collected samples, performed ELISA testing, and was involved in manuscript writing and edits, H.A. (Silent Heroes Foundation) designed experiments and arranged local logistics, A.O. (University of Tennessee) performed statistical analysis and was involved in manuscript writing and edits, and M.A.K. (University of Tennessee) was the project leader and was responsible for experimental design and data analysis.

Abstract

Background: Domestic dogs (*Canis familiaris*) have the potential to act as disease reservoirs for wildlife and are important sentinels for common circulating pathogens. Therefore, the infectious disease seroprevalence among domestic dogs in northern Botswana may be indicative of pathogen exposure of various wildlife species. The objective of this study was to assess the seroprevalence of *Ehrlichia spp.*, *Borrelia burgdorferi*, *Anaplasma spp.*, *Dirofilaria immitis*, canine adenovirus, canine parvovirus, and canine distemper virus in domestic dogs as proxies of disease prevalence in the local wildlife in the Okavango Delta region of Botswana. Statistical analysis assessed crude and factor-specific seroprevalence proportions and assessed the factors age, sex, and geographical location as predictors of seropositivity. Logistic regression was used to identify adjusted predictors of seropositivity for each of the pathogens of interest.

Results: Samples from 233 dogs in a total of 7 locations in Maun, Botswana, and surrounding villages were collected and serologically analyzed. No dogs were seropositive for *B. burgdorferi,* while low seroprevalence proportions were observed for *Anaplasma spp.* (2.2%) and *D. immitis* (0.9%). Higher seroprevalence proportions were observed for the tick-borne pathogen, *Ehrlichia spp.* (21.0%), and 19.7% were seropositive for canine adenovirus (hepatitis). The highest seroprevalence proportions were for canine parvovirus (70.0%) and canine distemper virus (44.8%). The predictors of seropositivity revealed that adults were more likely to be seropositive for canine adenovirus, canine distemper virus, and canine parvovirus than juveniles, and location was a risk factor for canine adenovirus, canine distemper virus, canine parvovirus, and *Ehrlichia spp*.

Conclusions: Results indicate that increasing tick control and vaccination campaigns for domestic dogs may improve the health of domestic animals, and potentially wildlife and humans in the Okavango Delta since viral and vector-borne bacterial pathogens can be transmitted between them.

Introduction

Vaccination of domestic dogs has been reported as a method of wildlife conservation [1] with the implication that prevalence of transmissible diseases in the domestic canine population has the potential to affect disease burden in wildlife, including both wild felids and wild canids. African wild dogs (*Lycaon pictus*), a wild canid species in sub-Saharan Africa, are endangered according to the International Union for the Conservation of Nature Redlist [2], and the black-footed cat (*Felis nigripes*), cheetah (*Acinonyx jubatus*), and lion (*Panthera leo*) are all vulnerable species in Botswana [2] that can be negatively impacted by domestic dog viral and vector-borne bacterial pathogens [3, 4, 5]. Capturing a sufficient number of African wild dogs, black-footed cats, cheetah, or lion to perform seroprevalence surveys is not always feasible due to the risk associated with anesthesia necessary to collect blood samples from these animals. This is particularly concerning due to the low numbers of individuals as indicated by their conservation status. McRee, et al. [6] performed a prevalence evaluation of viral pathogens in domestic dogs in northwest Zimbabwe as a representation of wildlife viral disease prevalence in the region, particularly African wild dogs.

Ehrlichia spp., *Anaplasma spp*., and *Borrelia burgdorferi* (Lyme disease) are bacterial pathogens that are transmitted by tick bites [7, 8, 9]. *Dirofilaria immitis*, or heartworm disease, is a blood-borne parasite transmitted by mosquito bites [10], and canine distemper (CDV),

parvovirus (CPV), and adenovirus (CAV) are viral diseases transmitted between individuals [11, 12, 13]. All of these common pathogens in dogs can cause serious illness and/or death. While the viral diseases, Lyme disease, and heartworm disease can be prevented either by vaccination or monthly heartworm preventative medication, many communities in southern Africa do not have the resources to pay for these medications for their animals. Thus, these preventable diseases may be widespread.

Botswana is a land-locked country in southern Africa and is home to the Okavango Delta, a diverse wetland habitat. Not only is the Okavango Delta home to countless species, it is the center for tourism in the country, which has become the second most important industry in Botswana after diamond mining [14]. The Okavango Delta of Botswana is rich with wildlife which have the chance to interact with domestic animal populations. This potentially results in cross-transmission of infections between domestic and wild animals implying that infectious disease exposure in domestic animals might mirror those of wildlife. As disease prevalence of common infectious diseases in wild carnivores is unknown in the Okavango Delta, this presents the opportunity to use domestic dogs as sentinels for infectious disease exposure in wildlife. Therefore, the objective of this study was to assess the seroprevalence of common infectious diseases (*Ehrlichia spp.*, *Borrelia burgdorferi*, *Anaplasma spp.*, *Dirofilaria immitis,* CDV, CAV, and CPV) in domestic dogs in Maun, Botswana, an area adjacent to the Okavango Delta, as a proxy for seroprevalence that would be expected in the wild canid and felid populations.

Materials and Methods

Animals

The University of Tenneessee Knoxville Intitutional Animal Care and Used Committee approved this research proposal on March 27, 2015; #2333-0315. All blood collections were done under the direct supervision of the veterinary members of the Maun Animal Welfare Society (MAWS). The majority (n=128/233) of blood samples from domestic dogs were collected using a convenience sampling strategy at the MAWS in Maun, Botswana, from dogs who were presented for castration and vaccination. The remaining blood samples (n=105/233) were collected in surrounding villages. The uncastrated domestic dogs in this area were highly unlikely to have been vaccinated and be seropositive to the viral diseases due to vaccination cross-reaction because MAWS is the main veterinary clinic in the area for the low income population, and they will not vaccinate animals unless they are also castrated at the time of vaccination. Therefore, animals that were reproductively intact are likely to be unvaccinated. Blood samples were collected from a peripheral vein, transported on ice, and stored at -20°C until testing.

Sample Analyses

Seroprevalence for CAV, CPV, and CDV were assessed using Biogal Titer Check (Biogal Galed Laboratories, Kibbutz Galed, Israel) per manufacturer instructions. The Biogal Titer Check results were reported with a change of color and ‘negative‘, ‘positive’, ‘highly positive’, or ‘inconclusive’ as the range of possible results. The vector-borne diseases, *Ehrlichia spp.*, *Borrelia burgdorferi*, *Anaplasma spp.*, and *Dirofilaria immitis*, were assessed with IDEXX 4DX SNAP ELISA (IDEXX, Westbrook, Maine, USA) per manufacturer instructions. The IDEXX SNAP ELISA results were reported as either ‘positive’ or ‘negative’ by a color change.

Statistical Analysis

Crude and factor-specific seroprevalence proportions of *Ehrlichia spp.*, *Borrelia burgdorferi*, *Anaplasma spp., Dirofilaria immitis*, CDV, CAV and CPV as well as their 95% exact confidence intervals were computed. The factors considered were age, sex, and location. Associations between seroprevalence and each of the above factors were assessed using the Chi-square or Fishers Exact tests as appropriate. Significance was set at $\alpha = 0.05$ for all statistical tests. Logistic regression was used to identify adjusted predictors of seropositivity for each of the pathogens of interest.

Results

Animal demographics

A total of 233 dogs were tested (Table 1). Females dogs made up the majority (54.5%) of the sampled dogs. Dogs were sampled during the months of June and July 2015 with the majority (55.4%) of the dogs being sampled in July. The age group distribution was 69.7% adults and 30.0% juveniles. Samples were collected at seven locations in Maun, Botswana, and surrounding villages: MAWS (n=128), Khumaga (n=13), Boro (n=18), Mathapane (n=16), Shorobe (n=18), Sexaxa (n=8), and Etsha (n=32).

Table 1: Characteristics of dogs included in a seroprevalence assessment of prior exposure to common pathogens in Botswana, 2015

Variable	Number	Percent	95% Exact Binomial Confidence Interval
Sex			
Female	127	54.5	47.9, 61.0
Male	106	45.5	3.90, 52.1
Age Category			

Adult	163	69.7	63.6, 75.8
Juvenile	70	30.0	24.2, 36.3
Location			
Boro	18	7.7	4.6, 11.9
Etsha	32	13.7	9.6, 18.8
Khumaga	13	5.6	3.0, 9.4
MAWS	128	54.9	48.3, 61.4
Mathapane	16	6.9	4.0, 10.9
Sexaxa	8	3.4	1.5, 6.7
Shorobe	18	7.7	4.6, 11.9
Month			
June 2015	104	44.6	38.1, 51.0
July 2015	129	55.4	48.7, 61.8

Crude seroprevalence

Of 232 individuals, 0% were seropositive for *Borrelia burgdorferi*, 2.2% were seropositive for *Anaplasma spp.*, and 0.9% were seropositive for *Dirofilaria immitis* (Table 2). Out of the 233 animals, 21.0% were seropositive for *Ehrlichia spp.*, 19.7% were seropositive for CAV, 70% were seropositive for CPV, and 46.8% were seropositive for CDV.

Table 2: Crude seroprevalence of selected pathogens among domestic dogs in Botswana, 2015

Pathogen	n	Number of seropositive samples	Percentage of seropositive samples	95% Confidence Interval
Anaplasma spp.	232	5	2.2	0.7, 5.0
B.burgdorferi	232	0	0	0, 1.6
D.immitis	232	2	0.9	0.1, 3.1
CDV	233	109	46.8	40.2, 53.4
Ehrlichia spp.	233	49	21.0	16.0, 26.8
CAV	233	46	19.7	14.8, 24.4
CPV	233	163	70.0	63.6, 75.8

Predictors of seropositivity

Based on the results of the logistic model, there were no statistically significant predictors (sex, age, month, or location) (P>0.05) for seropositivity of either *Anaplasma spp.* or *D. immitus* (Table 3).

Although age of the dog and location had significant unadjusted associations with the odds of CAV seropositivity, only age (OR=4.4; p<0.0003) was significant in the final; implying that the odds of CAV seropositivity was 4.4 times higher in adults dogs than in juveniles (Table 4). Similarly, only age had a significant association in the final model for CPV with adults having 4.4 times higher odds of CPV seropositivity (OR 4.4; p<0.0001) than juveniles (Table 4).

Based on the results of the logistic model, CDV seropositivity was significantly associated with age (p<0.0001), month (p=0.0002), and geographical location of sampling (p=0.0437) (Table 5). Although both age of the dog and geographical location had significant unadjusted association with the odds of *Ehrlichia spp.* seropositivity (Table 5) when both were offered to the model in a multivariable analysis, neither was significant. Therefore, there was no final multivariable model for *Ehrlichia spp.*

Controlling for the other two factors in the model, the odds of CDV seropositivity is 12 times higher among adult dogs than the juveniles (Table 6). Similarly, the odds of the dogs having seropositive results for CDV were 7.8 times higher in June than in July (Table 6). With respect to geographical location, only Khumaga (p=0.0014) and Shorobe (p=0.0481) had significantly different odds of canine seropositivity from MAWS with the odds of the dogs in Shorobe being 5.8 times higher than those of the reference group (MAWS) (Table 6). By contrast, the dogs in Khumaga had significantly lower odds (OR=0.072) of testing seropositive to CDV than dogs in MAWS. The odds of being seropositive for CDV among dogs from the other locations were not significantly different from that of MAWS. Based on the Hosmer-

Lemeshow Goodness-of-fit test, there is no evidence that the canine distemper model did not fit the data well (p=0.4813).

Table 3: Factor-specific seroprevalence of *Anaplasma spp.* and *D.immitis* among dogs in Botswana, 2015

**Pathogen**	**n**	**Number of seropositive samples**	**Percentage of seropositive samples**	**P-value**
Anaplasma spp.	**232**	**5**	**2.2**	
Sex				
Female	126	4	3.2	0.379
Male	106	1	0.9	
Age Category				
Adult	162	5	3.1	0.326
Juvenile	70	0	0	
Month				
June	104	1	1.0	0.383
July	128	4	3.1	
Location				
Boro	18	0	0	0.938
Etsha	32	0	0	
Khumaga	13	0	0	
MAWS	127	5	3.9	
Mathapane	16	0	0	
Sexaxa	8	0	0	
Shorobe	18	0	0	
D.immitis	**232**	**2**	**0.9**	
Sex				
Female	126	2	1.6	0.502
Male	106	0	0	
Age Category				
Adult	162	2	1.2	1.000
Juvenile	70	0	0	
Month				
June	104	1	1.0	1.000
July	128	1	0.8	
Location				
Boro	18	0	0	0.242
Etsha	32	0	0	
Khumaga	13	0	0	
MAWS	127	1	0.8	

Mathapane	16	0	0	
Sexaxa	8	1	12.5	
Shorobe	18	0	0	

Table 4: Factor-specific seroprevalence of CAV and CPV among dogs in Botswana, 2015

Pathogen	n	Number of seropositive samples	Percentage of seropositive samples	P-value
**CAV**	**233**	**46**	**19.7**	
Sex				
Female	127	20	15.6	0.101
Male	106	26	24.5	
Age Category				
Adult	163	41	25.2	0.001
Juvenile	70	5	7.1	
Month				
June	104	26	25.0	0.097
July	129	20	15.5	
Location				
Boro	18	6	33.3	0.043
Etsha	32	5	15.6	
Khumaga	13	2	15.4	
MAWS	128	22	17.2	
Mathapane	16	8	50	
Sexaxa	8	0	0	
Shorobe	18	3	16.7	
**CPV**	**233**	**163**	**70.0**	
Sex				
Female	127	92	72.4	0.392
Male	106	71	67.0	
Age Category				
Adult	163	130	79.8	<0.001
Juvenile	70	33	47.1	
Month				
June	104	78	75.0	0.152
July	129	85	65.9	
Location				
Boro	18	15	83.3	0.025
Etsha	32	16	50	
Khumaga	13	6	46.2	
MAWS	128	92	71.9	
Mathapane	16	12	75	
Sexaxa	8	6	75	
Shorobe	18	16	88.9	

Table 5: Factor-specific seroprevalence of CDV and *Ehrlichia spp.* among dogs in Botswana, 2015

Pathogen	n	Number of seropositive samples	Percentage of seropositive samples	P-value
**CDV**	**233**	**109**	**46.8**	
Sex				
Female	127	62	48.8	0.512
Male	106	47	44.3	
Age Category				
Adult	163	99	60.7	<0.001
Juvenile	70	10	14.3	
Month				
June	104	71	68.3	<0.001
July	129	38	29.5	
Location				
Boro	18	14	77.8	<0.001
Etsha	32	6	18.8	
Khumaga	13	2	15.4	
MAWS	128	51	39.8	
Mathapane	16	13	81.3	
Sexaxa	8	6	75.0	
Shorobe	18	17	94.4	
Ehrlichia spp.	**233**	**49**	**21.0**	
Sex				
Female	126	28	22.1	0.748
Male	106	21	19.8	
Age Category				
Adult	163	40	24.5	0.054
Juvenile	70	9	12.9	
Month				
June	104	22	21.2	1.000
July	129	27	20.9	
Location				
Boro	18	3	16.7	<0.001
Etsha	32	0	0	
Khumaga	13	0	0	
MAWS	128	40	31.3	
Mathapane	16	3	18.8	
Sexaxa	8	2	25	
Shorobe	18	1	7.6	

Table 6: Results of multivariable logistic regression showing predictors of CDV sero-positivity among domestic dogs in Botswana, 2019

Predictor	Odds Ratio	95% Confidence Interval	P-value
**Age**			
Adult	12.4	4.8, 32.1	<0.0001
Juvenile	Referent	Referent	
**Month**			
June	7.8	2.6, 23.1	0.0002
July	Referent	Referent	
**Location**			
Boro	0.7	0.2, 3.3	0.8022
Etsha	0.7	0.2, 1.9	0.7233
Khumaga	0.07	0.01, 0.5	0.0014
Mathapane	1.2	0.2, 6.5	0.5889
Sexaxa	1.2	0.2, 9.6	0.6601
Shorobe	5.8	0.6, 57.8	0.0481
MAWS	Referent	Referent	

Discussion

Domestic animals can serve as disease reservoirs for wildlife. Wild animal populations, including various canid and felid species, have the potential to be infected by CDV, CPV, CAV, *D. immitis, B. burgdorferi, Ehrlichia spp.* and *Anaplasma spp.,* which are pathogens carried by the domestic animal population. Free-ranging cheetah (*Acinonyx jubatus*) in Namibia have been reported to have antibodies to CPV (though this test cross-reacts with feline panleukopenia virus) and CDV [15]. A captive breeding group of African wild dogs in Tanzania showed 94% mortality after infection with CDV [16], and another group of African wild dogs in Kenya had higher disease-related mortality rates from 21% to 50% and CDV antibodies rose from 1-4% to 76% over a three-year period [3]. In Chobe National Park, Botswana, in 1996, a pack of twelve African wild dogs was reduced to two animals following an outbreak of CDV [17]. While the prevalence in the current study was low (0.9%), *D. immitis* can infect wildlife, with reports of *D.*

immitis in a captive lion in Spain [18] and a captive black-footed cat in Florida [19]. Butler, et al. [20] indicated that domestic dogs in northwest Zimbabwe are a source of disease transmission for leopards (*Panthera pardus*), lions, and spotted hyenas (*Crocuta crocuta*), as these predator species feed on domestic dogs as prey. A survey of domestic dogs and African Wild dogs in Kenya from 2001 to 2009 showed 16% of African wild dogs and 48% of domestic dogs had been exposed to CDV, 25% of African wild dogs and 64% of domestic dogs had been exposed to CPV, and 80% of African wild dogs and 86% of domestic dogs had been exposed to *E. canis* [21]. Similar to the Kenya evaluation, the results of the present study revealed 46.8% CDV seropositititivity and 70.0% CPV seropositity in domestic dogs. However, we found lower seropositivity rates for *Ehrlichia spp.* (21.0%) compared to the Kenya study.

When comparing regional infectious disease prevalence differences, similar seroprevalence for viral diseases in domestic dogs was determined in Botswana as was reported in a similar study performed in Zimbabwe in 2012 [6]. A study of domestic dogs in northwest Zimbabwe reported that 34% had antibodies to CDV, 84% had antibodies to CPV, and 13% had antibodies for CAV [6]. These results are similar to those of the present study of 46.8% for CDV, 70.0% for CPV, and 19.7% for CAV. Another seroprevalence study evaluating domestic dogs in northeast Namibia in 1993 and 1994 found similar exposure to CDV (44.3%), but lower prevalence of CPV (47.1%) and higher prevalence of CAV (64.3%) [22]. Both Zimbabwe and Namibia border Botswana on its eastern and western edges, respectively. Viral diseases spread from animal to animal so localized differences in exposure are expected, but vectored pathogens depend on prevalence of the pathogen, prevalence of the vector, and on contact between the vector and the susceptible animal host. Williams, et al. [23] assessed prevalence of several hemoparasites, including *Ehrlichia spp.*, in domestic dogs, lions, spotted hyena, and African wild

dogs in 2009 to 2011 in Zambia, another country in southern Africa that borders Botswana, and samples were evaluated by polymerase chain reaction which only reveals active infections rather than current and past exposure. No carnivores had positive results for *E. canis* or *E. ewingii* [23], which does not rule out presence of ehrlichia in the study area.

As ticks are the vectors for *B. burgdorferi, Ehrlichia spp.* and *Anaplasma spp*, tick prevalence is a crucial factor in the spread of these pathogens. While no recent studies have reported tick prevalence in northern Botswana, Eygelaar, *et al.* [24] reported that African buffalo (*Syncerus caffer*) in the Okavango Delta had lower prevalence of tick-borne diseases than African buffalo in Chobe National Park, a region in northeast Botswana that is closer to the study region assessed by McRee, et al. [6]. Perhaps the same cause is responsible for the reduced tick-vectored diseases in African buffalo and domestic dogs in the Okavango Delta compared to northwest Zimbabwe. Eygelaar, et al. [24] hypothesized that veterinary fences in the Okavango Delta prevented direct contact between the African buffalo and cattle, which were not present in Chobe National Park. Perhaps these same fences reduce tick spread from wildlife to domestic dogs and vice versa, which limits the spread of the vector-borne diseases in the Okavango Delta which were not present in northwest Zimbabwe. More research must be performed to determine if the differences in *Ehrlichia spp*. seroprevalence is due to a reduction in total tick numbers or another cause, such as physical barriers. The high viral disease seroprevalence, similar to those in northwest Zimbabwe, is likely due to lack of vaccination. While strong efforts are being actively put forth by local not-for-profit organizations, the number of unvaccinated dogs remains much greater than the number of vaccinated dogs.

Factor-specific seroprevalence indicated significant associations between seropositivity to these common canine infectious diseases and age, month, and location. Adults are more likely

to be seropositive for CAV, CDV, and CPV, which is likely due to having more time to be exposed to the viruses than juveniles. There was a significant association between seropositivity for CDV and month with June having higher risk than July. Lastly, geographical location is a risk factor for CAV, CDV, and CPV because viral pathogens are transmitted either by direct contact or contact with bodily fluids. Therefore, geographical locations with high rates of these pathogens allow easy transmission to naïve individuals. Location was also a risk factor for *E. canis* perhaps because of tick concentrations in certain locations or due to an increase in the pathogen in the dogs of certain locations that perpetuates the elevated infection rate (ticks can spread the pathogen transstadially, but not transovarially [7]) .

While the viral pathogens evaluated in this study cannot infect humans, some of the vectored pathogens can affect humans. In addition to affecting domestic and wild animal populations, *E. canis* and *E. ewingii* have been reported in humans [25, 26]. *B. burgdorferi*, the causative organism for Lyme disease and *A. phagocytophilum* [27, 28] are also zoonotic. The 'One Health' paradigm, a collaborative approach to animal, human, and environmental health that recognizes their interconnectivity, is particularly important, since reducing disease risk in domestic animals will reduce disease risk in wildlife and human populations. By increasing vaccination and reducing tick burden in domestic dogs, human health and environmental health, in the case of wildlife, are improved [29, 30].

One of the limitations of this study was that serology detects exposure to the pathogen, but it does not determine the rate of active infections. Thus, sero-prevalence indicates that pathogen exposure has occurred, but current risk of infection is unknown. This aspect is important for human and wildlife health because new infections may increase disease burden in these populations.

Information regarding disease prevalence is necessary to determine domestic animal, wildlife, and human disease risk. This study reveals the need for local tick surveys to determine the cause of tick-borne pathogen prevalence differences between Botswana and surrounding countries. In conclusion, further disease testing and vaccination of both domestic dogs and wildlife would benefit domestic animals, wildlife, and humans in the Okavango Delta region of Botswana.

Acknowledgements

We would like to thank all of the staff at the Maun Animal Welfare Society, including Tana, Justine, KC, and Nation. We would also like to thank Hansje de Waard, Deirdre Halloran, and Anna Hewitt for generous assistance in sample collection.

References

1. Cleaveland S, Kaare M, Knobel D, Laurenson MK. Canine vaccination—providing broader benefits for disease control. Vet microbiol. 2006 Oct 5;117(1):43-50.
2. International Union for Conservation of Nature. The IUCN Red List of Threatened Species; 2019 [cited 2019 May 13]. Database [Internet]. Available from: http://www.iucnredlist.org/
3. Alexander KA, Appel MJ. African wild dogs (Lycaon pictus) endangered by a canine distemper epizootic among domestic dogs near the Masai Mara National Reserve, Kenya. J Wildl Dis. 1994 Oct;30(4):481-485.

4. Roelke-Parker ME, Munson L, Packer C, Kock R, Cleaveland S, Carpenter M, et al. A canine distemper virus epidemic in Serengeti lions (Panthera leo). Nature. 1996 Feb;379(6564):441.
5. Steinel A, Parrish CR, Bloom ME, Truyen U. Parvovirus infections in wild carnivores. J Wildl Dis. 2001 Jul;37(3):594-607.
6. McRee A, Wilkes RP, Dawson J, Parry R, Foggin C, Adams H, Kennedy MA. Serological detection of infection with canine distemper virus, canine parvovirus and canine adenovirus in communal dogs from Zimbabwe. J S Afr Vet Assoc. 2014 Jan;85(1):01-2.
7. Groves MG, Dennis GL, Amyx HL, Huxsoll DL. Transmission of Ehrlichia canis to dogs by ticks (Rhipicephalus sanguineus). Am J Vet Res. 1975 Jul;36(7):937-940.
8. Stuen S. Anaplasma phagocytophilum-the most widespread tick-borne infection in animals in Europe. Vet Res Commun. 2007 Aug 1;31(1):79-84.
9. Johnson RC, Schmid GP, Hyde FW, Steigerwalt AG, Brenner DJ. Borrelia burgdorferi sp. nov.: etiologic agent of Lyme disease. Int J Syst Evol Microbiol. 1984 Oct 1;34(4):496-497.
10. GRIEVE RB, LOK JB, GLICKMAN LT. Epidemiology of canine heartworm infection. Epidemiol Rev. 1983 Jan 1;5(1):220-246.
11. Harder TC, Osterhaus AD. Canine distemper virus—a morbillivirus in search of new hosts?. Trends Microbiol. 1997 Mar 1;5(3):120-124.
12. Goddard A, Leisewitz AL. Canine parvovirus. Vet Clin North Am Small Anim Pract. 2010 Nov 1;40(6):1041-1053.

13. Decaro N, Martella V, Buonavoglia C. Canine adenoviruses and herpesvirus. Vet Clin North Am Small Anim Pract. 2008 Jul 1;38(4):799-814.
14. Mmopelwa G, Blignaut JN. The Okavango Delta. The value of tourism: economic. South African Journal of Economic and Management Sciences. 2006 Mar 1;9(1):113-127.
15. Munson L, Marker L, Dubovi E, Spencer JA, Evermann JF, O'Brien SJ. Serosurvey of viral infections in free-ranging Namibian cheetahs (Acinonyx jubatus). J Wildl Dis. 2004 Jan;40(1):23-31.
16. van de Bildt MW, Kuiken T, Visee AM, Lema S, Fitzjohn TR, Osterhaus AD. Distemper outbreak and its effect on African wild dog conservation. Emerg Infect Dis. 2002 Feb;8(2):212.
17. Alexander KA, Kat PW, Munson LA, Kalake A, Appel MJ. Canine distemper-related mortality among wild dogs (Lycaon pictus) in Chobe National Park, Botswana. J Zoo Wildl Med. 1996 Sep;27(3):426-427
18. De Ybanez MR, Martínez-Carrasco C, Martínez JJ, Ortiz JM, Attout T, Bain O. Dirofilaria immitis in an African lion (Panthera leo). Vet Rec. 2006 Feb 18;158(7):24
19. Deem SL, Heard DJ, LaRock R. Heartworm (Dirofilaria immitis) disease and glomerulonephritis in a black-footed cat (Felis nigripes). J Zoo Wildl Med. 1998 Jun 1;29:199-202.
20. Butler JR, Du Toit JT, Bingham J. Free-ranging domestic dogs (Canis familiaris) as predators and prey in rural Zimbabwe: threats of competition and disease to large wild carnivores. Biol Conserv. 2004 Feb 1;115(3):369-378.

21. Woodroffe R, Prager KC, Munson L, Conrad PA, Dubovi EJ, Mazet JA. Contact with domestic dogs increases pathogen exposure in endangered African wild dogs (Lycaon pictus). PLoS One. 2012 Jan 6;7(1):e30099.
22. Laurenson K, Van Heerden J, Stander P, Van Vuuren MJ. Seroepidemiological survey of sympatric domestic and wild dogs (Lycaon pictus) in Tsumkwe District, northeastern Namibia. Onderstepoort J Vet Res. 1997 Dec 1;64:313-316.
23. Williams BM, Berentsen A, Shock BC, Teixiera M, Dunbar MR, Becker MS, et al. Prevalence and diversity of Babesia, Hepatozoon, Ehrlichia, and Bartonella in wild and domestic carnivores from Zambia, Africa. Parasitol Res. 2014 Mar 1;113(3):911-918.
24. Eygelaar D, Jori F, Mokopasetso M, Sibeko KP, Collins NE, Vorster I, et al. Tick-borne haemoparasites in African buffalo (Syncerus caffer) from two wildlife areas in Northern Botswana. Parasit Vectors. 2015 Dec;8(1):26.
25. Conrad ME. Ehrlichia canis: a tick-borne rickettsial-like infection in humans living in the southeastern United States. Am J Med Sci. 1989 Jan 1;297(1):35-37.
26. Buller RS, Arens M, Hmiel SP, Paddock CD, Sumner JW, Rikihisa Y, et al. Ehrlichia ewingii, a newly recognized agent of human ehrlichiosis. N Engl J Med. 1999 Jul 15;341(3):148-155.
27. Murphree Bacon R, Kugeler KJ, Mead PS. Surveillance for Lyme disease--United States, 1992-2006. 2008.
28. Dumler JS, Choi KS, Garcia-Garcia JC, Barat NS, Scorpio DG, Garyu JW, et al. Human granulocytic anaplasmosis and Anaplasma phagocytophilum. Emerg Infect Dis. 2005 Dec;11(12):1828.

29. Day MJ. One health: the importance of companion animal vector-borne diseases. Parasit Vectors. 2011 Dec;4(1):49.

30. Dantas-Torres F, Chomel BB, Otranto D. Ticks and tick-borne diseases: a One Health perspective. Trends Parasitol. 2012 Oct 1;28(10):437-446.

Click here to access/download
Supporting Information
MAWS_Constitution 2010.pdf

Click here to access/download
Supporting Information
Kennedy 2333-0315 final.docx

Click here to access/download
Supporting Information
Maun Canine Data 2.xlsx

Click here to access/download
Supporting Information
BotswanaCanineData_Submitted_to_PLOSONE.xls

Seroprevalence of viral and vector~~f~~-borne ~~various~~ pathogens in domestic dogs (*Canis familiaris)* in northern Botswana

Short title: ~~Infectious disease~~ P~~p~~revalence ~~-~~of antibodies to i~~I~~nfectious agents ~~in dogs~~ in Botswanan dogs

Riley E. Thompson[1], Hayley Adams[2], Agricola Odoi[3], Melissa A. Kennedy[3]

[1]Department of Large Animal Clinical Sciences, College of Veterinary Medicine, University of Tennessee, Knoxville, TN, USA, [2]Silent Heroes Foundation, USA, [3]Department of Biomedical and Diagnostic Sciences, College of Veterinary Medicine, University of Tennessee, Knoxville, TN, USA

Correspondence to:

Melissa Kennedy

Email:

mkenned2@utk.edu

Author contributions

R.E.T. (University of Tennessee) collected samples, performed ELISA testing, and was involved in manuscript writing and edits, H.A. (Silent Heroes Foundation) designed experiments and arranged local logistics, A.O. (University of Tennessee) performed statistical analysis and was

involved in manuscript writing and edits, and M.A.K. (University of Tennessee) was the project leader and was responsible for experimental design and data analysis.

Abstract

Background: Domestic dogs (*Canis familiaris*) have the potential to act as disease reservoirs for wildlife and are important sentinels for common circulating pathogens. Therefore, the infectious agent ~~disease~~ seroprevalence among domestic dogs in northern Botswana may be indicative of pathogen exposure of various wildlife species. The objective of this study was to assess the seroprevalence of *Ehrlichia* spp., *Borrelia burgdorferi*, *Anaplasma* spp., *Dirofilaria immitis*, canine adenovirus, canine parvovirus-2, and canine distemper virus in domestic dogs as proxies of disease prevalence in the local wildlife in the Okavango Delta region of Botswana. Statistical analysis assessed crude and factor-specific seroprevalence proportions and assessed the factors age, sex, and geographical location as predictors of seropositivity. Logistic regression was used to identify adjusted predictors of seropositivity for each of the pathogens of interest.

Results: Samples from 233 dogs in ~~a total of~~ 7 locations in Maun, Botswana, and surrounding villages were collected and serologically analyzed. No dogs were seropositive for *B. burgdorferi*, while low seroprevalence proportions were observed for *Anaplasma* spp. (2.2%) and *D. immitis* (0.9%). Higher seroprevalence proportions were observed for the tick-borne pathogen, *Ehrlichia* spp. (21.0%), and 19.7% were seropositive for canine adenovirus (hepatitis). The highest seroprevalence proportions were for canine parvovirus-2 (70.0%) and canine distemper virus (44.8%). The predictors of seropositivity revealed that adults were more likely to be seropositive for canine adenovirus, canine distemper virus, and canine parvovirus-2 than

Formatted: Font: Not Italic

Formatted: Font: Not Italic

Formatted: Font: Not Italic

Formatted: Font: Not Italic

juveniles, and location was a risk factor for canine adenovirus, canine distemper virus, canine parvovirus-2, and *Ehrlichia* spp.

Formatted: Font: Not Italic

Conclusions: Results ~~indicate~~ indicate a need for tick prevalence data and that increasing ~~tick control and~~ vaccination campaigns for domestic dogs may improve the health of domestic animals, and potentially wildlife and humans in the Okavango Delta since viral and vector-borne bacterial pathogens can be transmitted between them.

Introduction

Vaccination of domestic dogs has been reported as a method of wildlife conservation [1] with the implication that prevalence of transmissible diseases in the domestic canine population has the potential to affect disease burden in wildlife, including both wild felids and wild canids. African wild dogs (*Lycaon pictus*), a wild canid species in sub-Saharan Africa, are endangered according to the International Union for the Conservation of Nature Redlist [2], and the black-footed cat (*Felis nigripes*), cheetah (*Acinonyx jubatus*), and lion (*Panthera leo*) are all vulnerable species in Botswana [2] that can be negatively impacted by domestic dog viral and vector-borne bacterial pathogens [3-~~, 4, ~~5]. Capturing a sufficient number of these wild canids and felids~~African wild dogs, black-footed cats, cheetah, or lion~~ to perform seroprevalence surveys is not always feasible due to the risk- associated with anesthesia necessary to collect blood samples from these animals. This is particularly concerning due to the low numbers of individuals as indicated by their conservation status. McRee, et al. [6] performed a seroprevalence study~~evaluation~~ of viral pathogens in domestic dogs in northwest Zimbabwe as a representation of wildlife viral disease agent prevalence in the region, particularly in African wild dogs.

Ehrlichia spp., *Anaplasma* spp., and *Borrelia burgdorferi* (Lyme disease) are bacterial pathogens that are transmitted by tick bites [7, 8, 9]. *Dirofilaria immitis*, or heartworm disease, is a blood-borne parasite transmitted by mosquito bites [10], and canine distemper virus (CDV), parvovirus-2 (CPV2), and adenovirus (CAV) are viral diseases transmitted between individuals [11, 12, 13]. All of these common pathogens in dogs can cause serious illness and/or death. While the viral diseases, Lyme disease, and heartworm disease can be prevented either by vaccination or monthly heartworm preventative medication, many communities in southern Africa do not have the resources to pay for these medications for their animals. Thus, these preventable diseases may be widespread.

Botswana is a land-locked country in southern Africa and is home to the Okavango Delta, a diverse wetland habitat. Not only is the Okavango Delta home to countless species, it is the center for tourism in the country, which has become the second most important industry in Botswana after diamond mining [14]. The Okavango Delta of Botswana is rich with wildlife which have the chance to interact with domestic animal populations. This potentially results in cross-transmission of infections between domestic and wild animals implying that infectious disease exposure in domestic animals might mirror those of wildlife. As disease prevalence of common infectious diseases in wild carnivores is unknown in the Okavango Delta, this presents the opportunity to use domestic dogs as sentinels for infectious disease exposure in wildlife. Therefore, the objective of this study was to assess the seroprevalence of common infectious disease agentss (*Ehrlichia* spp., *Borrelia burgdorferi*, *Anaplasma* spp., *Dirofilaria immitis*, CDV, CAV, and CPV2) in domestic dogs in Maun, Botswana, an area adjacent to the Okavango Delta, as a proxy for seroprevalence that would be expected in the wild canid and felid populations.

Formatted: Font: Not Italic

Formatted: Font: Not Italic

Formatted: Font: Not Italic

Formatted: Font: Not Italic

Our hypothesis is that these agents will be present at significant levels in domestic dogs indicating their presence in this geographic locale.

Materials and Methods

Animals

~~All blood collections were done under the direct supervision of the veterinary members of the Maun Animal Welfare Society (MAWS).~~ The majority (n=128/233) of blood samples from domestic dogs were collected using a convenience sampling strategy at the Maun Animal Welfare Society (MAWS) in Maun, Botswana, from dogs who were presented for castration and vaccination and under the direct supervision of the staff veterinarian. The remaining blood samples (n=105/233) were collected in surrounding villages also under veterinary supervision. Informed consent was obtained verbally from each owner of dogs sampled. The uncastrated domestic dogs in this area were highly unlikely to have received any vaccines except rabies vaccination ~~been vaccinated~~ and be seropositive to the viral diseases due to vaccination cross-reaction. This is because MAWS is the main veterinary clinic in the area for the low income population, and they will not vaccinate animals unless they are also castrated at the time of vaccination. Therefore, animals that were reproductively intact are likely to be unvaccinated. Demographic data was recorded at the time of sample collection including sex, breed, and approximate age of 'juvenile' or 'adult' based on dentition and overall appearance. Blood samples of 1-2ml volume were collected from a peripheral vein, transported on ice, and whole blood stored at -4~~20~~°C until testing within 48 hours.

Sample Analyses

Formatted: Strikethrough

One to two ml of whole blood were collected in an EDTA ~~red top~~ tube from 233 ~~(232?)~~ dogs. ~~Centrifugation was done and serum samples were collected.~~ Seroprevalence for CAV-, CPV2-, and CDV-specific antibodies were assessed using Biogal ~~Titer Check~~ImmunoComb Canine VacciCheck (Biogal Galed Laboratories, Kibbutz Galed, Israel) per manufacturer instructions. The Biogal Titer Check results were reported with a change of color and 'negative' (no color development), 'positive' (equal color development to the reference spot- 1:16 VN for CAV, 1:80 HI for CPV2, and 1:32 VN for CDV), 'highly positive' (color development greater than the reference spot), or 'inconclusive' (very faint color development) as the range of possible results. The vector-borne diseases, *Ehrlichia* spp., *Borrelia burgdorferi*, *Anaplasma* spp., and *Dirofilaria immitis*, were assessed with IDEXX 4DX SNAP ELISA (IDEXX, Westbrook, Maine, USA) per manufacturer instructions. The IDEXX SNAP ELISA results were reported as either 'positive' or 'negative' by a color change. Rabies seroprevalence was not assessed.

Statistical Analysis

All statistical a~~A~~nalyses were performed in SAS (Statistical Analysis Systems). Crude and factor-specific seroprevalence proportions of *Ehrlichia* spp., *Borrelia burgdorferi*, *Anaplasma* spp., *Dirofilaria immitis*, CDV, CAV and CPV as well as their 95% exact confidence intervals were computed. The factors considered were age, sex, and location. Associations between seroprevalence and each of the above factors were assessed using the Chi-square or Fishers Exact tests as appropriate. Significance was set at α = 0.05 for all statistical tests. Logistic regression was used to identify adjusted predictors of seropositivity for each of the pathogens of interest.

Formatted: Font: Not Italic

Formatted: Font: Not Italic

Formatted: Font: Not Italic

Formatted: Font: Not Italic

Results

Animal demographics

A total of 233 dogs were tested (Table 1). However, at least one of the dogs had missing data and so some of the statistical analyses were performed on only 232 records. Females dogs made up the majority (54.5%) of the sampled dogs. Dogs were sampled during the months of June and July 2015 with the majority (55.4%) of the dogs being sampled in July. The age group distribution was 69.7% adults and 30.0% juveniles. ~~Serum~~ Whole blood samples were collected at seven locations in Maun, Botswana, and surrounding villages: MAWS (n=128), Khumaga (n=13), Boro (n=18), Mathapane (n=16), Shorobe (n=18), Sexaxa (n=8), and Etsha (n=32). All dogs were of mixed breed. Rabies seroprevalence studies were~~was~~ not assessed as vaccination status was unknown, and no point-of-care ELISA or similar was available. The latter would have been necessary since canine serum could not be transported for testing.

Table 1: Characteristics of dogs included in a seroprevalence assessment of prior exposure to common pathogens in Botswana, 2015

Variable (n=233)	Number	Percent	95% Exact Binomial Confidence Interval
Sex			
Female	127	54.5	47.9, 61.0
Male	106	45.5	3.90, 52.1
Age Category			
Adult	163	69.7	63.6, 75.8
Juvenile	70	30.0	24.2, 36.3
Location			
Boro	18	7.7	4.6, 11.9
Etsha	32	13.7	9.6, 18.8
Khumaga	13	5.6	3.0, 9.4
MAWS	128	54.9	48.3, 61.4
Mathapane	16	6.9	4.0, 10.9
Sexaxa	8	3.4	1.5, 6.7
Shorobe	18	7.7	4.6, 11.9
Month			
June 2015	104	44.6	38.1, 51.0
July 2015	129	55.4	48.7, 61.8

Crude seroprevalence

Of 232 ~~individuals~~dogs tested for *Borrelia burgdorferi*, *Anaplasma* spp and *Dirofilaria*, 0% were seropositive for *Borrelia burgdorferi*, 2.2% were seropositive for *Anaplasma spp.*, and 0.9% were seropositive for *Dirofilaria immitis* (Table 2). One dog had missing data for the above 3 pathogens. Out of the 233 ~~animals~~dogs, 21.0% were seropositive for *Ehrlichia spp.*, 19.7% were seropositive for CAV, 70% were seropositive for CPV2, and 46.8% were seropositive for CDV.

Formatted: Font: Italic

Table 2: Crude seroprevalence of selected pathogens among domestic dogs in Botswana, 2015

Pathogen	Number of seropositive samples	Percentage of seropositive samples	95% ~~Confidence Interval~~CI1
Anaplasma spp.(n=232)*	5	2.2	0.7, 5.0
B.burgdorferi (n=232)*	0	0	0, 1.6
D.immitis (n=232)*	2	0.9	0.1, 3.1
~~C~~Distemper Virus (n=233)	109	46.8	40.2, 53.4
Ehrlichia spp. (n=233)	49	21.0	16.0, 26.8
~~C~~Adenovirus~~V~~ (n=233)	46	19.7	14.8, 24.4
~~CPV~~Parvovirus (n=233)	163	70.0	63.6, 75.8

* There was missing data for the pathogens *Anaplasma*, *B.burgdorferi*, and *D.immitis* and so the sample size was 232 and not 233
95% CI: 95% confidence interval

Predictors of seropositivity

Based on univariable analyses~~the results of the logistic model~~, none of the variables assessed (sex, age, month, or location) had a ~~there were no~~ statistically significant (p>0.05) ~~predictors (sex, age, month, or location) (P>0.05) for~~association with seropositivity of either *Anaplasma* spp. or *D. immitus* (Table 3a) ~~(Table 3a)~~. Although both age of the dog and geographical location had significant unadjusted association with *Ehrlichia* spp. seropositivity (Table 3b), when both were offered to the model in a multivariable analysis, only age was significant (p=0.0846) (Table 4).

~~Although~~Both age of the dog and location had significant unadjusted associations with the odds of CAV seropositivity, only age (OR=4.4; p<0.0003) was significant in the final; implying that the odds of CAV seropositivity was 4.4 times higher in adult~~s~~ dogs than in juveniles (Table 4). Similarly, only age had a significant association in the final model for CPV

Formatted: Line spacing: single

Formatted Table

Formatted: Font: Not Italic

Formatted: Line spacing: single

Formatted: Font: Italic

Formatted: Font: Italic

Formatted: Font: Italic

Formatted: Font: Not Italic

Formatted: Font: Not Italic

Formatted: Indent: First line: 0.5"

with adults also having 4.4 times higher odds of CPV2 seropositivity (OR=~~=~~4.4; p<0.0001) than juveniles (Table 4).

For CDV, the final ~~Based on the results of the~~ logistic model~~,~~ showed that CDV seropositivity was significantly associated with age (p<0.0001), month (p=0.0002), and geographical location of sampling (p=0.0437) (Table ~~5~~4). ~~Although both age of the dog and geographical location had significant unadjusted association with the odds of *Ehrlichia* spp. seropositivity (Table 5) when both were offered to the model in a multivariable analysis, neither was significant. Therefore, there was no final multivariable model for *Ehrlichia* spp.~~

Controlling for the other two factors in the model, the odds of CDV seropositivity ~~is~~ was 12 times higher among adult dogs than ~~the~~ juveniles (Table 4~~6~~). Similarly, the odds of the dogs having seropositive results for CDV were 7.8 times higher in June than in July (Table 4~~6~~). With respect to geographical location, only Khumaga (p=0.0014) and Shorobe (p=0.0481) had significantly different odds of canine seropositivity from MAWS with the odds of seropositivity for CDV among ~~the~~ dogs in Shorobe being 5.8 times higher than those of the reference group (MAWS) (Table 4~~6~~). By contrast, the dogs in Khumaga had significantly lower odds (OR=0.07~~2~~) of testing seropositive to CDV than dogs in MAWS. The odds of being seropositive for CDV among dogs from the other locations were not significantly different from that of MAWS. Based on the Hosmer-Lemeshow Goodness-of-fit test, there is no evidence that the canine distemper model did not fit the data well (p=0.4813).

~~Based on the Hosmer-Lemeshow Goodness-of-fit test, there is no evidence that the canine distemper model did not fit the data well (p=0.4813).~~

Formatted: Not Highlight

Formatted: Not Highlight

Formatted: Font: Not Italic

Formatted: Font: Not Italic

Formatted: Not Highlight

Formatted: Level 1

Table 3a: Factor-specific seroprevalence of ~~Anaplasma spp. and D.immitis~~selected pathogens among dogs in Botswana, 2015

	Anaplasma spp. (n=232)				*D.immitis (n=232)*				Adenovirus (n=233)				Parvovirus (n=233)			
Pathogen	n[2]~~n~~	# pos[1] ~~Number of seropositive samples~~	~~Percentage~~ % pos[1] ~~of seropositive samples~~	~~P-value~~p[3]	n[2]	# pos[1]	% pos[1]	p[3]	n[2]	# pos[1]	% pos[1]	p[3]	n[2]	# pos[1]	% pos[1]	p[3]
Total~~Anaplasma spp.~~	232	5	2.2		232	2	0.9		233	46	19.7		233	163	70.0	
Sex				0.379				0.502								
Female	126	4	3.2	~~0.379~~	126	2	1.6		127	20	15.6	0.101	127	92	72.4	0.392
Male	106	1	0.9		106	0	0		106	26	24.5		106	71	67.0	
Age Category				0.326				1.000								
Adult	162	5	3.1	~~0.326~~	162	2	1.2		163	41	25.2	0.001	163	130	79.8	<0.001
Juvenile	70	0	0		70	0	0		70	5	7.1		70	33	47.1	
Month				0.383				1.000								
June	104	1	1.0	~~0.383~~	104	1	1.0		104	26	25.0	0.097	104	78	75.0	0.152
July	128	4	3.1		128	1	0.8		129	20	15.5		129	85	65.9	
Location				0.938				0.242								
Boro	18	0	0	~~0.938~~	18	0	0		18	6	33.3	0.043	18	15	83.3	0.025
Etsha	32	0	0		32	0	0		32	5	15.6		32	16	50	
Khumaga	13	0	0		13	0	0		13	2	15.4		13	6	46.2	
MAWS	127	5	3.9		127	1	0.8		128	22	17.2		128	92	71.9	
Mathapane	16	0	0		16	0	0		16	8	50		16	12	75	
Sexaxa	8	0	0		8	1	12.5		8	0	0		8	6	75	
Shorobe	18	0	0		18	0	0		18	3	16.7		18	16	88.9	

[1]Positive
[2]Number of samples tested (note that there is missing data for *Anaplasma* and *D.immitis* and so there total samples is 232 instead of 233)
[3]p-value

Table 3b: Factor-specific seroprevalence of selected pathogens among dogs in Botswana, 2015

	Distemper spp. (n=233)				Ehrlichia (n=233)			
Pathogen	n[2]	# pos[1]	% pos[1]	p[3]	n[2]	# pos[1]	% pos[1]	p[3]
Total	233	109	46.8		233	49	21.0	
Sex								
Female	127	62	48.8	0.512	126	28	22.1	0.748
Male	106	47	44.3		106	21	19.8	
Age Category								
Adult	163	99	60.7	<0.001	163	40	24.5	0.054
Juvenile	70	10	14.3		70	9	12.9	
Month								
June	104	71	68.3	<0.001	104	22	21.2	1.000
July	129	38	29.5		129	27	20.9	
Location								
Boro	18	14	77.8	<0.001	18	3	16.7	<0.001
Etsha	32	6	18.8		32	0	0	
Khumaga	13	2	15.4		13	0	0	
MAWS	128	51	39.8		128	40	31.3	
Mathapane	16	13	81.3		16	3	18.8	
Sexaxa	8	6	75.0		8	2	25	
Shorobe	18	17	94.4		18	1	7.6	

[1]Positive
[2]Number of samples tested (note that there is missing data for *Anaplasma* and *D.immitis* and so there total samples is 232 instead of 233)
[3]p-value

~~Table 4: Factor-specific seroprevalence of CAV and CPV among dogs in Botswana, 2015~~

~~Pathogen~~	~~n~~	~~Number of seropositive samples~~	~~Percentage of seropositive samples~~	~~P-value~~
~~CAV~~	~~233~~	~~46~~	~~19.7~~	
~~Sex~~				
~~Female~~	~~127~~	~~20~~	~~15.6~~	~~0.101~~
~~Male~~	~~106~~	~~26~~	~~24.5~~	
~~Age Category~~				
~~Adult~~	~~163~~	~~41~~	~~25.2~~	~~0.001~~
~~Juvenile~~	~~70~~	~~5~~	~~7.1~~	
~~Month~~				
~~June~~	~~104~~	~~26~~	~~25.0~~	~~0.097~~
~~July~~	~~129~~	~~20~~	~~15.5~~	
~~Location~~				
~~Boro~~	~~18~~	~~6~~	~~33.3~~	~~0.043~~
~~Etsha~~	~~32~~	~~5~~	~~15.6~~	
~~Khumaga~~	~~13~~	~~2~~	~~15.4~~	
~~MAWS~~	~~128~~	~~22~~	~~17.2~~	
~~Mathapane~~	~~16~~	~~8~~	~~50~~	
~~Sexaxa~~	~~8~~	~~0~~	~~0~~	
~~Shorobe~~	~~18~~	~~3~~	~~16.7~~	
~~CPV~~	~~233~~	~~163~~	~~70.0~~	
~~Sex~~				

Formatted Table
Formatted: Font: 9 pt
Formatted Table
Formatted: Font: 9 pt
Formatted: Font: 9 pt
Formatted: Font: 9 pt
Formatted: Font: 9 pt
Formatted: Font: 9 pt
Formatted: Font: 9 pt
Formatted: Font: 9 pt
Formatted: Font: 9 pt
Formatted: Font: 9 pt
Formatted: Font: 9 pt
Formatted: Font: 9 pt
Formatted: Font: 9 pt
Formatted: Font: 9 pt
Formatted: Font: 9 pt
Formatted: Font: 9 pt
Formatted: Font: 9 pt
Formatted: Font: 9 pt
Formatted: Font: 9 pt
Formatted: Font: 9 pt

Female	127	92	72.4	0.392
Male	106	71	67.0	
Age Category				
Adult	163	130	79.8	<0.001
Juvenile	70	33	47.1	
Month				
June	104	78	75.0	0.152
July	129	85	65.9	
Location				
Boro	18	15	83.3	0.025
Etsha	32	16	50	
Khumaga	13	6	46.2	
MAWS	128	92	71.9	
Mathapane	16	12	75	
Sexaxa	8	6	75	
Shorobe	18	16	88.9	

Table 5: Factor-specific seroprevalence of CDV and *Ehrlichia spp.* among dogs in Botswana, 2015

Pathogen	n	Number of seropositive samples	Percentage of seropositive samples	P-value
CDV	233	109	46.8	
Sex				
Female	127	62	48.8	0.512
Male	106	47	44.3	
Age Category				
Adult	163	99	60.7	<0.001
Juvenile	70	10	14.3	
Month				
June	104	71	68.3	<0.001
July	129	38	29.5	
Location				
Boro	18	14	77.8	<0.001
Etsha	32	6	18.8	
Khumaga	13	2	15.4	
MAWS	128	51	39.8	
Mathapane	16	13	81.3	
Sexaxa	8	6	75.0	
Shorobe	18	17	94.4	
Ehrlichia spp.	233	49	21.0	
Sex				
Female	126	28	22.1	0.748
Male	106	21	19.8	
Age Category				
Adult	163	40	24.5	0.054
Juvenile	70	9	12.9	
Month				
June	104	22	21.2	1.000
July	129	27	20.9	
Location				
Boro	18	3	16.7	<0.001
Etsha	32	0	0	
Khumaga	13	0	0	

218

~~MAWS~~	~~128~~	~~40~~	~~31.3~~
~~Mathapane~~	~~16~~	~~3~~	~~18.8~~
~~Sexaxa~~	~~8~~	~~2~~	~~25~~
~~Shorobe~~	~~18~~	~~1~~	~~7.6~~

Formatted: Font: 9 pt

Formatted: Font: 9 pt

Formatted: Font: 9 pt

Formatted: Font: 9 pt

Table 4~~6~~: Results of multivariable logistic regression showing predictors of CDV sero-positivity among domestic dogs in Botswana, 2019

	Canine Adenovirus Model			Canine Parvovirus Model			Canine Di~~y~~stemper Virus Model			Erhlichia canis Model		
**Predictor**	OR[1] ~~Odds Ratio~~	95% CI[2]~~95% Confidence Interval~~	P-value~~P-value~~	OR[1]	95% CI[2]	P-value	OR[1]	95% CI[2]	P-value	OR[1]	95% CI[2]	P-value
**Age**			0.0031			<0.0001			<0.0001			0.0486
Adult	4.4~~1 2.4~~	1.6,11.6~~4 .8, 32.1~~	0.0031~~< 0.0001~~	4.4	2.4, 8.1	<0.0001	12.4	4.8, 32.1	<0.0001	2.2	1.0, 4.8	0.0486
Juvenile	Ref[3] ~~Referent~~	Ref[3]~~Referent~~		Ref[3]	Ref[3]		Ref[3]	Ref[3]				
**Month**	NS[4]			NS[4]					0.0002	NS[4]		
June	~~7.8~~	~~2.6, 23.1~~	~~0.0002~~				7.8	2.6, 23.1	0.0002			
July	~~Referent~~	~~Referent~~					Ref[3]	Ref[3]				
**Location**	NS[4]			NS[4]					0.0437	NS[4]		
Boro	~~0.7~~	~~0.2, 3.3~~	~~0.8022~~				0.7	0.2, 3.3	0.8022			
Etsha	~~0.7~~	~~0.2, 1.9~~	~~0.7233~~				0.7	0.2, 1.9	0.7233			
Khumaga	~~0.07~~	~~0.01, 0.5~~	~~0.0014~~				0.07	0.01, 0.5	0.0014			
Mathapane	~~1.2~~	~~0.2, 6.5~~	~~0.5889~~				1.2	0.2, 6.5	0.5889			
Sexaxa	~~1.2~~	~~0.2, 9.6~~	~~0.6601~~				1.2	0.2, 9.6	0.6601			
Shorobe	~~5.8~~	~~0.6, 57.8~~	~~0.0481~~				5.8	0.6, 57.8	0.0481			
MAWS	~~Referent~~	~~Referent~~					Ref[3]	Ref[3]				

[1]Odds Ratio
[2]95% Confidence Interval
[3]Reference Group
[4]Not statistically significant (p>0.05) and therefore not included in the final model

Discussion

Domestic animals can serve as disease reservoirs for wildlife. Wild animal populations, including various canid and felid species, have the potential to be infected by CDV, CPV2, CAV, *D. immitis*, *B. burgdorferi*, *Ehrlichia* spp. and *Anaplasma* spp., which are pathogens carried by the domestic animal population. Free-ranging cheetah (*Acinonyx jubatus*) in Namibia have been reported to have antibodies to CPV2 (though this test cross-reacts with feline panleukopenia virus) and CDV [15]. A captive breeding group of African wild dogs in Tanzania showed 94% mortality after infection with CDV [16], and another group of African wild dogs in Kenya had higher disease-related mortality rates from 21% to 50% and CDV antibodies rose from 1-4% to 76% over a three-year period [3]. In Chobe National Park, Botswana, in 1996, a pack of twelve African wild dogs was reduced to two animals following an outbreak of CDV [17]. While the prevalence in the current study was low (0.9%), *D. immitis* can infect wildlife, with reports of *D. immitis* in a captive lion in Spain [18] and a captive black-footed cat in Florida [19]. Butler, et al. [20] indicated that domestic dogs in northwest Zimbabwe are a source of disease transmission for leopards (*Panthera pardus*), lions, and spotted hyenas (*Crocuta crocuta*), as these predator species feed on domestic dogs as prey. A survey ~~of domestic dogs and African Wild dogs~~ in Kenya from 2001 to 2009 showed 16%, 25%, and 80% of African wild dogs and 48%, 64% and 86% of domestic dogs had been exposed to CDV, ~~25% of African wild dogs and 64% of domestic dogs had been exposed to~~ CPV2, and ~~80% of African wild dogs and 86% of domestic dogs had been exposed to~~ *E. canis* respectively [21]. Similar to the Kenya evaluation, the results of the present study revealed 46.8% CDV seropostitivity and 70.0% CPV2 seropositiv~~tity~~y in domestic dogs. However, we found lower seropositivity rates for *Ehrlichia* spp. (21.0%) compared to the Kenya study.

Formatted: Font: Not Italic

Formatted: Font: Not Italic

Formatted: Font: Not Italic

When comparing regional infectious disease prevalence differences, similar seroprevalence for viral diseases in domestic dogs was determined in Botswana as was reported in a similar study performed in Zimbabwe in 2012 [6]. A study of domestic dogs in northwest Zimbabwe reported that 34% had antibodies to CDV, 84% had antibodies to CPV2, and 13% had antibodies for CAV [6]. These results are similar to those of the present study of 46.8% for CDV, 70.0% for CPV2, and 19.7% for CAV. Another seroprevalence study evaluating domestic dogs in northeast Namibia in 1993 and 1994 found similar exposure to CDV (44.3%), but lower prevalence of CPV2 (47.1%) and higher prevalence of- CAV (64.3%) [22]. Both Zimbabwe and Namibia border Botswana on its eastern and western edges, respectively. Viral diseases spread from animal to animal so localized differences in exposure are expected, but vectored pathogens depend on prevalence of the pathogen, prevalence of the vector, and on contact between the vector and the susceptible animal host. Williams, et al. [23] assessed prevalence of several hemoparasites, including *Ehrlichia* spp., in domestic dogs, lions, spotted hyena, and African wild dogs in 2009 to 2011 in Zambia, another country in southern Africa that borders Botswana, and samples were evaluated by polymerase chain reaction which only reveals active infections rather than current and past exposure. No carnivores had positive results for *E. canis* or *E. ewingii* [23], which does not rule out presence of ehrlichia in the study area.

Formatted: Font: Not Italic

As ticks are the vectors for *B. burgdorferi, Ehrlichia spp*. and *Anaplasma spp*, tick prevalence is a crucial factor in the spread of these pathogens. - *Ixodes* spp. are the vectors for *B. burgdorferi*, and *Rhipicephalus* spp. are the primary vectors for *E. canis* and *Anaplasma* spp., although other tick species have been reported to transmit both pathogens. While no recent studies have reported tick prevalence in northern Botswana, Eygelaar, *et al.* [24] reported that African buffalo (*Syncerus caffer*) in the

Okavango Delta had lower prevalence of tick-borne diseases than African buffalo in Chobe National Park, a region in northeast Botswana that is closer to the study region assessed by McRee, et al. [6]. Perhaps the same cause is responsible for the reduced tick-vectored diseases in African buffalo and domestic dogs in the Okavango Delta compared to northwest Zimbabwe. Eygelaar, et al. [24] hypothesized that veterinary fences in the Okavango Delta prevented direct contact between the African buffalo and cattle, which were not present in Chobe National Park. Perhaps these same fences reduce tick spread from wildlife to domestic dogs and vice versa, which limits the spread of the vector-borne diseases in the Okavango Delta which were not present in northwest Zimbabwe. More research must be performed to determine if the differences in *Ehrlichia* spp. seroprevalence is due to a reduction in total tick numbers or another cause, such as physical barriers. The high viral disease seroprevalence, similar to those in northwest Zimbabwe, is likely due to lack of vaccination. While strong efforts are being actively put forth by local not-for-profit organizations, the number of unvaccinated dogs remains much greater than the number of vaccinated dogs.

Formatted: Font: Not Italic

Factor-specific seroprevalence indicated significant associations between seropositivity to these common canine infectious diseases and age, month, and location. Adults are more likely to be seropositive for CAV, CDV, and CPV2, which is likely due to having more time to be exposed to the viruses than juveniles. There was a significant association between seropositivity for CDV and month with June having higher risk than July. The reason for this is unclear and will require further investigation in future studies. Lastly, geographical location is a risk factor for CAV, CDV, and CPV2 because viral pathogens are transmitted either by direct contact or contact with bodily fluids. Therefore, geographical locations with high rates of these pathogens allow easy transmission to naïve individuals. Location was also a risk factor for *E. canis* perhaps because of tick concentrations in certain

locations or due to an increase in the pathogen in the dogs of certain locations that perpetuates the elevated infection rate (ticks can spread the pathogen tran~~s~~stadially, but not transovarially [7]) .

While the viral pathogens evaluated in this study cannot infect humans, some of the vectored pathogens can affect humans. In addition to affecting domestic and wild animal populations, *E. canis* and *E. ewingii* have been reported in humans [25, 26]. *B. burgdorferi*, the causative organism for Lyme disease, and *A. phagocytophilum* [27, 28] are also zoonotic. The 'One Health' paradigm, a collaborative approach to animal, human, and environmental health that recognizes their interconnectivity, is particularly important, since reducing disease risk in domestic animals will reduce disease risk in wildlife and human populations. By increasing vaccination ~~and reducing tick burden~~ in domestic dogs, human health and environmental health, in the case of wildlife, are improved [29, 30].

One of the limitations of this study was that serology detects exposure to the pathogen, but it does not determine the rate of active infections. Thus, sero-prevalence indicates that pathogen exposure has occurred, but current risk of infection is unknown. This aspect is important for human and wildlife health because new infections may increase disease burden in these populations.

Conclusions

Formatted: Font: Bold

Information regarding disease prevalence is necessary to determine domestic animal, wildlife, and human disease risk. This study reveals the need for local tick surveys to determine the cause of tick-borne pathogen prevalence differences between

Botswana and surrounding countries. In conclusion, further disease testing and vaccination of both domestic dogs and wildlife would benefit domestic animals, wildlife, and humans in the Okavango Delta region of Botswana.

Acknowledgements

We would like to thank all of the staff at the Maun Animal Welfare Society, including Tana, Justine, KC, and Nation. We would also like to thank Hansje de Waard, Deirdre Halloran, and Anna Hewitt for generous assistance in sample collection.

References

1. Cleaveland S, Kaare M, Knobel D, Laurenson MK. Canine vaccination—providing broader benefits for disease control. Vet microbiol. 2006 Oct 5;117(1):43-50.
2. International Union for Conservation of Nature. The IUCN Red List of Threatened Species; 2019 [cited 2019 May 13]. Database [Internet]. Available from: http://www.iucnredlist.org/
3. Alexander KA, Appel MJ. African wild dogs (Lycaon pictus) endangered by a canine distemper epizootic among domestic dogs near the Masai Mara National Reserve, Kenya. J Wildl Dis. 1994 Oct;30(4):481-485.
4. Roelke-Parker ME, Munson L, Packer C, Kock R, Cleaveland S, Carpenter M, et al. A canine distemper virus epidemic in Serengeti lions (Panthera leo). Nature. 1996 Feb;379(6564):441.

5. Steinel A, Parrish CR, Bloom ME, Truyen U. Parvovirus infections in wild carnivores. J Wildl Dis. 2001 Jul;37(3):594-607.

6. McRee A, Wilkes RP, Dawson J, Parry R, Foggin C, Adams H, Kennedy MA. Serological detection of infection with canine distemper virus, canine parvovirus and canine adenovirus in communal dogs from Zimbabwe. J S Afr Vet Assoc. 2014 Jan;85(1):01-2.

7. Groves MG, Dennis GL, Amyx HL, Huxsoll DL. Transmission of *Ehrlichia canis* to dogs by ticks (*Rhipicephalus sanguineus*). Am J Vet Res. 1975 Jul;36(7):937-940.

8. Stuen S. *Anaplasma phagocytophilum*-the most widespread tick-borne infection in animals in Europe. Vet Res Commun. 2007 Aug 1;31(1):79-84.

9. Johnson RC, Schmid GP, Hyde FW, Steigerwalt AG, Brenner DJ. *Borrelia burgdorferi* sp. nov.: etiologic agent of Lyme disease. Int J Syst Evol Microbiol. 1984 Oct 1;34(4):496-497.

10. Grieve~~RIEVE~~ RB, Lok~~OK~~ JB, Glickman~~LICKMAN~~ LT. Epidemiology of canine heartworm infection. Epidemiol Rev. 1983 Jan 1;5(1):220-246.

11. Harder TC, Osterhaus AD. Canine distemper virus—a morbillivirus in search of new hosts?. Trends Microbiol. 1997 Mar 1;5(3):120-124.

12. Goddard A, Leisewitz AL. Canine parvovirus. Vet Clin North Am Small Anim Pract. 2010 Nov 1;40(6):1041-1053.

Formatted: Font: Italic

Formatted: Font: Italic

Formatted: Font: Italic

Formatted: Font: Italic

13. Decaro N, Martella V, Buonavoglia C. Canine adenoviruses and herpesvirus. Vet Clin North Am Small Anim Pract. 2008 Jul 1;38(4):799-814.

14. Mmopelwa G, Blignaut JN. The Okavango Delta. The value of tourism: economic. South African Journal of Economic and Management Sciences. 2006 Mar 1;9(1):113-127.

15. Munson L, Marker L, Dubovi E, Spencer JA, Evermann JF, O'Brien SJ. Serosurvey of viral infections in free-ranging Namibian cheetahs (Acinonyx jubatus). J Wildl Dis. 2004 Jan;40(1):23-31.

16. van de Bildt MW, Kuiken T, Visee AM, Lema S, Fitzjohn TR, Osterhaus AD. Distemper outbreak and its effect on African wild dog conservation. Emerg Infect Dis. 2002 Feb;8(2):212.

17. Alexander KA, Kat PW, Munson LA, Kalake A, Appel MJ. Canine distemper-related mortality among wild dogs (Lycaon pictus) in Chobe National Park, Botswana. J Zoo Wildl Med. 1996 Sep;27(3):426-427

18. De Ybanez MR, Martínez-Carrasco C, Martínez JJ, Ortiz JM, Attout T, Bain O. *Dirofilaria immitis* in an African lion (Panthera leo). Vet Rec. 2006 Feb 18;158(7):24

19. Deem SL, Heard DJ, LaRock R. Heartworm (*Dirofilaria immitis*) disease and glomerulonephritis in a black-footed cat (*Felis nigripes*). J Zoo Wildl Med. 1998 Jun 1;29:199-202.

20. Butler JR, Du Toit JT, Bingham J. Free-ranging domestic dogs (*Canis familiaris*) as predators and prey in rural Zimbabwe: threats of competition and disease to large wild carnivores. Biol Conserv. 2004 Feb 1;115(3):369-378.

Formatted: Font: Italic

Formatted: Font: Italic

Formatted: Font: Italic

Formatted: Font: Italic

21. Woodroffe R, Prager KC, Munson L, Conrad PA, Dubovi EJ, Mazet JA. Contact with domestic dogs increases pathogen exposure in endangered African wild dogs (*Lycaon pictus*). PLoS One. 2012 Jan 6;7(1):e30099.

22. Laurenson K, Van Heerden J, Stander P, Van Vuuren MJ. Seroepidemiological survey of sympatric domestic and wild dogs (*Lycaon pictus*) in Tsumkwe District, northeastern Namibia. Onderstepoort J Vet Res. 1997 Dec 1;64:313-316.

23. Williams BM, Berentsen A, Shock BC, Teixiera M, Dunbar MR, Becker MS, et al. Prevalence and diversity of *Babesia*, *Hepatozoon*, *Ehrlichia*, and *Bartonella* in wild and domestic carnivores from Zambia, Africa. Parasitol Res. 2014 Mar 1;113(3):911-918.

24. Eygelaar D, Jori F, Mokopasetso M, Sibeko KP, Collins NE, Vorster I, et al. Tick-borne haemoparasites in African buffalo (*Syncerus caffer*) from two wildlife areas in Northern Botswana. Parasit Vectors. 2015 Dec;8(1):26.

25. Conrad ME. *Ehrlichia canis*: a tick-borne rickettsial-like infection in humans living in the southeastern United States. Am J Med Sci. 1989 Jan 1;297(1):35-37.

26. Buller RS, Arens M, Hmiel SP, Paddock CD, Sumner JW, Rikihisa Y, et al. *Ehrlichia ewingii*, a newly recognized agent of human ehrlichiosis. N Engl J Med. 1999 Jul 15;341(3):148-155.

27. Murphree Bacon R, Kugeler KJ, Mead PS. Surveillance for Lyme disease--United States, 1992-2006. 2008.

28. Dumler JS, Choi KS, Garcia-Garcia JC, Barat NS, Scorpio DG, Garyu JW, et al. Human granulocytic anaplasmosis and *Anaplasma phagocytophilum*. Emerg Infect Dis. 2005 Dec;11(12):1828.

29. Day MJ. One health: the importance of companion animal vector-borne diseases. Parasit Vectors. 2011 Dec;4(1):49.

30. Dantas-Torres F, Chomel BB, Otranto D. Ticks and tick-borne diseases: a One Health perspective. Trends Parasitol. 2012 Oct 1;28(10):437-446.
