# Supplementary material for: Seroprevalence of viral and vector-borne bacterial pathogens in domestic dogs (Canis familiaris) in northern Botswana
Source: PLoS One. 2020 Jan 8;15(1):e0220593. doi: 10.1371/journal.pone.0220593 (PMC6949005; doi:10.1371/journal.pone.0220593)
Supplement: Attachment — Submitted filename: Cor PONE Rev 1.docx [file pone.0220593.s004.docx]

Dear Editor

I have read through the revised manuscript PONE-D-19-20072. The MS has improved except for some few typo errors (highlighted on the ms) which the authors are advised to correct. My major concern, however, is the use of the word “seroprevalence” both in the title and the body of the text considering the fact that the authors used whole blood and not serum (permit my ignorance, if am off key) for the antibody detection. Once that is sorted out, the MS can be accepted for publication and I do not need to see it again

Regards

Joshua Kamani
