# Supplementary material for: Seroprevalence of viral and vector-borne bacterial pathogens in domestic dogs (Canis familiaris) in northern Botswana
Source: PLoS One. 2020 Jan 8;15(1):e0220593. doi: 10.1371/journal.pone.0220593 (PMC6949005; doi:10.1371/journal.pone.0220593)
Supplement: Attachment — Submitted filename: Response to Reviewers (minor revisions).doc [file pone.0220593.s005.doc]

November 25, 2019

To the Editor:

All of the reviewer comments were addressed and can be found in the track changes version. Per the comments regarding the tables, changes were made as outlined below:

Table 2. Why is there a difference in number tested for Anaplasma, Borrelia and Dinofilaria compared to the others?

Response:

1 record in the dataset had missing information for the three agents. I have added a footnote to table 2 to explain this.

**Reviewer comments:**

Table 3, 4 and 5‐ Is it possible to fill in some of the gaps (even a hyphen may help), or merge cells?

Response:

It is unclear which gaps the reviewer is referring to because there are really no gaps on tables 3,4 and 5. The p-value column, for instance, must have only one entry per variable. So the blank cells must remain the way they are (no not need to have any entries)

Additionally, the rows that have the variable (factor) names were formatted to not have any additional text to make it easy to read the table. I hope this explanation helps.

We believe the comments made and used strengthen this manuscript. We offer this revised manuscript for your deliberation.

Thank you for your consideration.

Sincerely,

Melissa Kennedy, Corresponding Author

**Reviewers’ comments:**

Laboratory methods – given that the only techniques used involved ELISA kits per manufacturers’ recommendation, none were deposited.

The style requirements were reviewed.

Owners’ verbal consent for participation, the locale of participants, and the volume of blood collected were addressed in the text.

*Reviewer #1:*

The short title was modified per the reviewer’s recommendations.

The italics for “spp.” designations were removed.

“total of” was removed from the sentence in line 37.

The issue of tick infestation and control were addressed in the text

Lines 60-61 and line 64 were modified per the reviewer’s recommendations.

The tick vector for each organism was included.

Rephrasing of lines 87-89 was made.

As stated in the manuscript, rabies was not assessed because of unknown vaccination status for rabies, and the lack of a point-of-care assay for this assessment.

The various aspects of demographic data were addressed in the text.

The use of whole blood was addressed

The various properties of the kits were addressed.

Color change evaluation was addressed.

Tables were condensed.

Lines 202-206 were modified per the reviewer’s recommendation.

The reference corrections were made.

*Reviewer #2:*

The hypothesis, objectives and methods were clarified.

Demographic data was addressed.

The discussion section was adjusted.

Tables were reduced:

**Reviewer Comment**

Line 134: is it 232 or 233?

**Response**

The total sample size was 233. However, there was one missing data point for test results of the following pathogens: Anaplasma spp., B.burgdorferi and D.immitis. Due to that missing data, these pathogens had 232 instead of 233. The rest of the pathogens did not have missing data and therefore had a total of 233. These explanations have been added as footnotes on tables 2, 3a and 3b

**Reviewer Comment**

Table 2: I am not sure the authors explained why there are two populations; 232 233 being tested for different pathogens.

**Response**

There is only one population of dogs. However, as explained above there was there was one missing data point for test results for Anaplasma spp., B.burgdorferi and D.immitis. Therefore these pathogens had data for only 232 dogs instead of 233 that the rest of the pathogen had.

**Reviewer Comment**

Table 3: The table can be presented in a better format without having to repeat the variables in columns 1 and 2 for the two pathogens. Why are some variables in column 1 in italics? (see throughout the tables)

**Response**

We thank the reviewer for their suggestion on improving the presentation of our results. As per the suggestion, tables 3, 4 and 5 have now been combined into Table 3. However, since the new Table 3 cannot fit on one page, it has been split into Table 3a and Table 3b. This change has allowed us to stop repeating information from column 1. However, we have retained column 2 because the data changes between pathogens depending on whether there was missing data or not.

**Reviewer Comment**

Tables 4 and 5 should be merged similar to comments made for table 3 to avoid duplicating the variables in columns 1 and 2.

Response:

As stated above tables 3, 4 and 5 have not been combined into Table 3. Since the Table 3 cannot fit on one page, it has been split into Table 3a and Table 3b. This change has allowed us to stop repeating information from column 1. However, we have retained column 2 because the data changes between pathogens depending on whether there was missing data or not.

**Reviewer Comment**

Line 240-41: What could influence the significant association of CDV with June than July? The study duration of 2 months to me is too short to make any meaningful deduction other than chance findings.

**Response:**

We thank the reviewer for noting this association and seeking an explanation. Unfortunately, this being a sero-survey, the scope of the study was limited and so we were not able to investigate this association further. However, due to the very strong association (OR = 7.8; p=0.002), it is unlikely to be due to chance. Suffice it to say that future studies will need to investigate this association further. We have added this comment in the manuscript.

Reference corrections were made.
